# Theoretical Investigation of the Circularly Polarized Luminescence of a Chiral Boron Dipyrromethene (BODIPY) Dye

**DOI:** 10.3389/fchem.2020.00801

**Published:** 2020-09-15

**Authors:** Qin Yang, Marco Fusè, Julien Bloino

**Affiliations:** Scuola Normale Superiore, Pisa, Italy

**Keywords:** circularly polarized luminescence, vibronic spectroscopy, chirality, BODIPY, mode mixing, Franck-Condon, electronic transition current density

## Abstract

Over the last decade, molecules capable of emitting circularly polarized light have attracted growing attention for potential technological and biological applications. The efficiency of such devices depend on multiple parameters, in particular the magnitude and wavelength of the peak of emitted light, and also on the dissymmetry factor for chiral applications. In light of these considerations, molecular systems with tunable optical properties, preferably in the visible spectral region, are particularly appealing. This is the case of boron dipyrromethene (BODIPY) dyes, which exhibit large molecular absorption coefficients, have high fluorescence yields, are very stable, both thermally and photochemically, and can be easily functionalized. The latter property has been extensively exploited in the literature to produce chromophores with a wide range of optical properties. Nevertheless, only a few chiral BODIPYs have been synthetized and investigated so far. Using a recently reported axially chiral BODIPY derivative where an axially chiral BINOL unit has been attached to the chromophore unit, we present a comprehensive computational protocol to predict and interpret the one-photon absorption and emission spectra, together with their chiroptical counterparts. From the physico-chemical properties of this molecule, it will be possible to understand the origin of the circularly polarized luminescence better, thus helping to fine-tune the properties of interest. The sensitivity of such processes require accurate results, which can be achieved through a proper account of the vibrational structure in optical spectra. Methodologies to compute vibrationally-resolved electronic spectra can now be applied on relatively large chromophores, such as BODIPYs, but require more extensive computational protocols. For this reason, particular attention is paid in the description of the different steps of the protocol, and the potential pitfalls. Finally, we show how, by means of appropriate tools and approaches, data from intermediate steps of the simulation of the final spectra can be used to obtain further insights into the properties of the molecular system under investigation and the origin of the visible bands.

## 1. Introduction

Circularly polarized luminescence (CPL) processes have gained interest over the years for their potential use in technological applications, such as data storage or optical displays, as well as for the design of novel biological probes (Riehl and Richardson, 1986; Furumi, 2010; Carr et al., 2012; Kumar et al., 2015; Sánchez-Carnerero et al., 2015; Zinna and Di Bari, 2015; Longhi et al., 2016; Jimènez et al., 2017; Li et al., 2018). A significant hurdle in passing from the proof of concept to potential industrial applications is the emitted signal. Besides the problem of performance, suitable candidates must meet a number of criteria in terms of stability, production cost, and toxicity. A relatively straightforward strategy is to start with a promising core structure, whose physico-chemical properties can be tuned through chemical substitutions or additions.

Among such tunable systems, Boron dipyrrin (4-bora-3a,4a-diaza-*s*-indacene, BODIPY) derivatives represent an important family of organic molecules that have shown excellent performance in photonic applications, such as 3D optical displays, storage, spintronics, or biological probes (Durán-Sampedro et al., 2013; Lifschitz et al., 2013; Wang et al., 2013; Liu et al., 2014; Zhao et al., 2015; Kaur and Singh, 2019; Turksoy et al., 2019), thanks to their absorption and emission properties in the visible spectra region, which can be easily manipulated (Loudet and Burgess, 2007; Ulrich et al., 2008; Wang et al., 2013; Liu et al., 2014; Zhao et al., 2015). They are also very stable and highly soluble in an extensive range of common organic solvents, and have the advantage of being economically more affordable and less hazardous than other light-emitting complexes based on rare earth or radioactive atoms (Kamkaew et al., 2013; Shivran et al., 2016; Zhang et al., 2020). These features make them appealing for the development of OLED devices or biological probes, for instance. While for the latter, the importance of chirality is well-established, interesting possibilities brought by the use of chiroptical signals in displays have also been proposed. However, most BODIPYs are achiral, so they do not exhibit any circular dichroism (CD) or CPL signals. For this reason, research groups have been exploring the possibility of synthetizing chiral BODIPYs, by adding chiral substituents or by tweaking the structure to be intrinsically chiral (Sánchez-Carnerero et al., 2014; Zinna et al., 2016; Alnoman et al., 2016; Lu et al., 2016; Jimènez et al., 2017; Abbate et al., 2017; Pop et al., 2019). The broad structural possibilities can pose a significant challenge in identifying the most suitable candidates. The inherent complexity of such a task can be reduced with the assistance of computational chemistry, which can help understanding the physico-chemical properties at the origin of the observed phenomena, and rationalize the chemical design. Thanks to hardware and software improvements, quantum chemical calculations can now be routinely done on molecular systems of increasing size and complexity, encompassing a large number of BODIPYs. Nevertheless, computational cost and accuracy still need to be carefully balanced, and the definition of a suitable computational protocol can be a complex task in view of the many methods available. The difficulty is further aggravated by the sensitivity of chiroptical spectroscopies, so that common models, which may be sufficient for non-chiral properties, may fall short, being unable to describe properly the properties involved and the radiative processes. This begins from the choice of the electronic structure calculation method, but also includes the representation of the potential energy surfaces and their vibrational structures. The latter is often disregarded but plays an essential role in determining the shape and intensity of absorption and emission spectra (Le Guennic et al., 2012; Pedone et al., 2012; Avila Ferrer et al., 2013; Barone et al., 2014; Hodecker et al., 2016; Liu et al., 2016; Padula et al., 2016; Hu et al., 2017; Fortino et al., 2019). With these considerations in mind, an extensive study of the methods available to describe excited-states properties of molecular systems of this size can be highly valuable. Through a description and illustration of their capabilities, it will be possible to design a comprehensive, but also evolutive, protocol for the study of chiroptical spectra of medium-to-large molecular systems beyond the standard, purely electronic methods.

**Figure 1:**
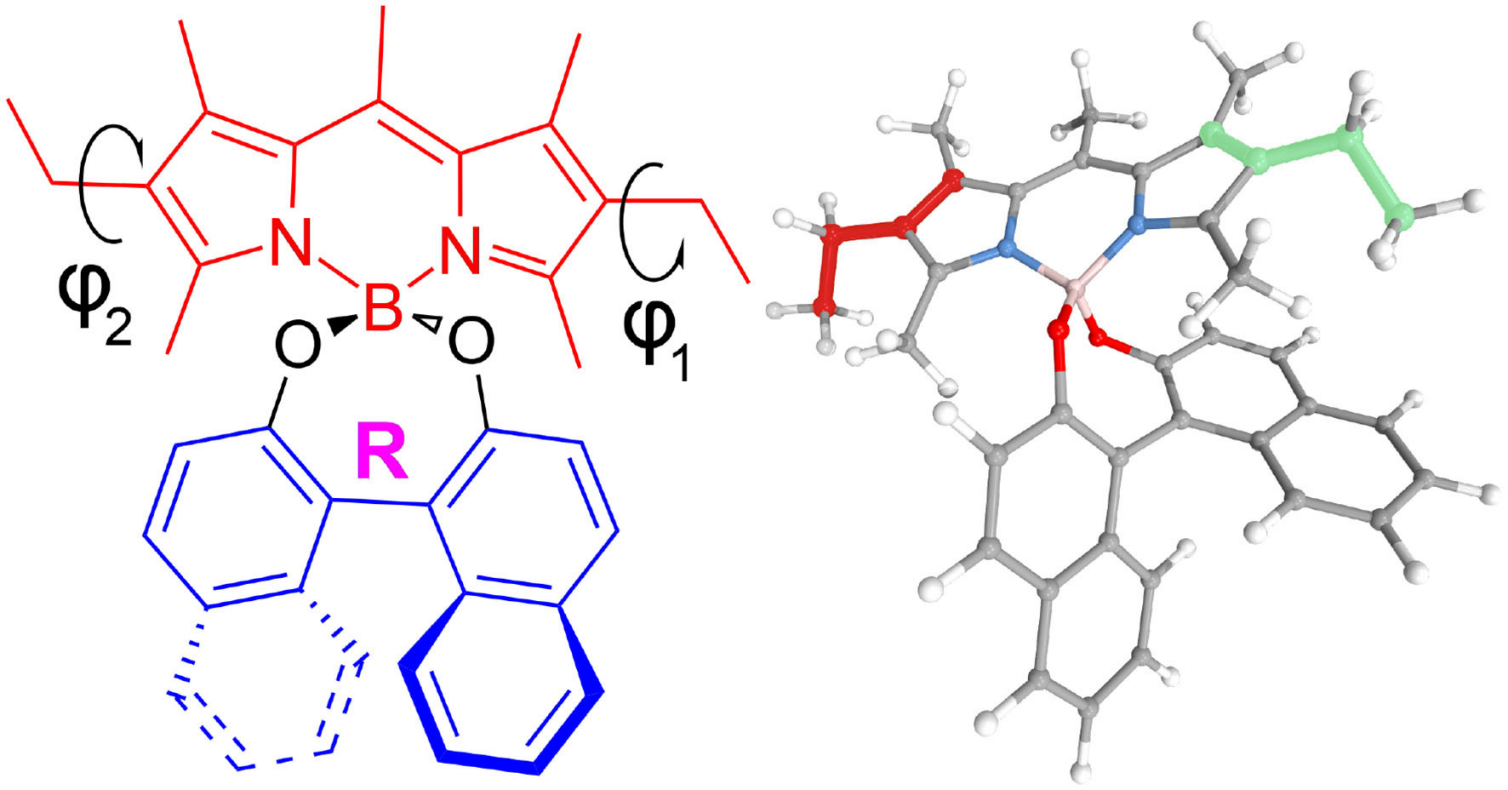
2D and 3D representations of (*R*)-O-BODIPY. ϕ_1_ (red in the 3D representation) and ϕ_2_ (green) represent the torsion angles for the scan.

As a test subject, we have chosen a derivative of BODIPY, called O-BODIPY by the original authors, where an axially chiral 1,1′-binaphthyl unit is orthogonally attached to the O-BODIPY chromophore, which was recently synthetized and studied experimentally (Sánchez-Carnerero et al., 2014; Gartzia-Rivero et al., 2017; Jiménez et al., 2019). Electronic circular dichroism (ECD) and CPL were used to characterize the chiroptical properties of such a design, shown in Figure 1. Both provide complementary information to the standard UV-visible absorption and fluorescence spectroscopies to get a comprehensive picture of the excited states of those molecules. Despite the low dissymmetry ratio (*g*_*lum*_(λ) = 2(*I*_*L*_(λ) − *I*_*R*_(λ))/(*I*_*L*_(λ) + *I*_*R*_(λ)), with *I*_*L*_ and *I*_*R*_ the left- and right-circularly polarized emitted lights, respectively), an interesting property of this system is to show a sign inversion between ECD and CPL for the lowest-energy band. This phenomenon was speculated to be connected to the presence of an intramolecular charge transfer before the emission. With the aim of characterizing the nature of this process and understand the chiroptical properties of this chiral O-BODIPY, we present an extensive computational protocol, starting from the definition of the electronic structure calculation method based on their overall performance up to the simulation of the vibrationally-resolved electronic spectra. We will introduce available methodologies rooted in the time-independent formalism to simulate the latter, with a discussion on their strengths and possible pitfalls. The whole procedure is sustained by suitable graphical representations, with intermediate data generated during the simulations providing valuable information to check the reliability of the results and get further insights into the properties of the system (Licari et al., 2015; Fusè et al., 2019).

The manuscript is organized as follows. In the first part, the main concepts used for the analysis of the results will be recalled and summarized. The discussion will be done as an incremental process, starting from the identification of conformers and their relative abundances, followed by the simulation of spectra with a growing level of sophistication. At each point, the reliability and potential caveats will be studied. Finally, following the protocol identified to be performing the best, an in-depth vibrational analysis will be carried out, with an analysis of the most relevant data produced by the vibronic simulation, which can shed light on structural optimization to enhance the properties of those systems.

## 2. Methods

### 2.1. General Overview

Expressed in general terms, the simulation of spectra will require two steps, the proper definition of the sample, to match experimental conditions and setup, and the choice of a suitable level of theory for the modeling of the response of the sample itself. Regarding the former, solvent can play an important role and, in case of direct interactions with the solute or if the solvent molecules have a significant impact on the overall spectra, must be treated explicitly, with the inclusion of solvent molecules. In the present case, polarizable continuum models, which reproduce the overall electrostatic properties of the solvent, are sufficient and were used. With a pure sample, the main issue in the modeling is the possible presence of conformers, which requires an investigation of the so-called conformational space, that is, the ensemble of all structures reachable by structural deformations with no bond breaking. Topological search or dynamics-based samplings are commonly used but can be time-consuming. For semi-rigid systems, like this is the case here, the actual degrees of freedoms are limited and a more directed search, based on chemical intuition is often more efficient.

It should be noted that the identification of stable conformers require a sufficient level of theory, which can be far lower than what needs to be employed for the simulation of spectra. With medium-large molecules like the ones of interest here, a trade-off between accuracy and computational cost is unavoidable. Density functional theory (DFT) remains the method of choice for such applications, with the well-known problem of identifying the best-suited functional. The standard approach is thus through a benchmark, which can be reduced to a small subset, based on existing knowledge accumulated in the literature. In the present case, because the target is not pure electronic spectra, but more sophisticated approaches, the problem of error compensation during this step must be kept in mind. Hence, instead of a best-performing functional for a given spectrum, it is more important to choose a well-balanced method, from which the potential energy surface and the transition moments of the properties of interest will be defined. The next subsections present the overall computational details for standard calculations, with emphasis on the methodological aspects related to the less common vibronic calculations.

### 2.2. Computational Details

All computations were done with a locally modified version of the Gaussian suite of quantum chemical programs, able to handle internal coordinates in vibronic calculations (Frisch et al., 2019). Methods rooted in DFT—for the electronic ground state—and its time-dependent extension (TD-DFT)—for the excited states—have been employed as they represent the most suitable choice in terms of efficiency and accuracy for systems of this size. For all calculations, the 6-31+G(d) basis set was employed, except where specified otherwise, and an ultrafine grid (99 radial shells and 590 angular points per shell) was used to integrate the exchange-correlation kernel. To match experimental condition, solvent effects due to the presence of chloroform were included by means of the polarizable continuum model in its integral equation formalism (IEF-PCM) (Cancès et al., 1997). The solute cavity was built as a combination of interlocking spheres centered on each atom with a diameter equal to their van der Waals radii scaled by a factor of 1.1. Equilibrium structures were reached using tight convergence criteria (maximum forces and displacements smaller than 1.5 × 10^−5^ Hartree/Bohr and 6 × 10^−5^ Å, respectively) for the geometry optimization of the ground electronic states, and loose criteria (1.67 × 10^−3^ Hartree/Bohr and 1.6 × 10^−3^ Å) for excited states, the latter being chosen to provide a balance between computational cost and accuracy. The quality of the “loose” convergence was checked for the first singlet excited state of one of the conformers of (*R*)-O-BODIPY (PC, see the next section for details) in terms of geometrical changes and frequencies. Maximum and mean deviations of 2.7 × 10^−2^ and 4 × 10^−3^ Å for the geometry, and 1.7 and 0.1 cm^−1^ for the vibrations were found. Analytic frequencies are available for both ground and excited states and were used to check that the true minima had been obtained.

The electronic transition current densities (ETCDs) were computed with a locally modified version of the Cubegen utility of Gaussian and saved as a discretized volumetric dataset in plain-text cube files. 3D ETCD figures representing the vector field were obtained as described in Fusè et al. (2019). Space partitioning within the QTAIM (Quantum theory of atoms in molecules) theory was done with the Multiwfn package (Lu and Chen, 2012) and volumetric datasets were processed with Python scripts.

For all spectra, Gaussian distribution functions were used to simulate the natural broadening observed in experimental spectra. In most cases, the value of the half-width at half-maximum was chosen to match the reference band-shape.

### 2.3. Vibronic Calculations

For vibrationally resolved electronic spectra, also referred to vibronic spectra in the following, the protocol extensively described in Bloino et al. (2016) was followed. We will just summarize here the most relevant aspects. Two complementary formalisms were adopted here. The sum-over-state approach (or time-independent, TI) was used to check the convergence of the spectrum and obtain information on the origin of the bands where relevant. The former represents the ratio between the calculated intensity obtained by summing the contributions from the individual transitions between each populated initial state and the manifold of final vibrational states, and the total intensity calculated by applying analytic sum rules. A low value, caused by the contribution of an exceedingly large number of low-intensity transitions, is often indicative of a strong mixing or displacement of the vibrational modes in conjunction with the electronic transition. This is due to significant structural changes, which may hint at a potential breakdown of the underlying Franck-Condon principle. Indeed, the theory used here assumes that the system is sufficiently rigid and the electronic transition induces relatively small geometrical deformations. The path-integral (or time-dependent, TD) formalism provides converged band-shapes, in presence of temperature effects as well. Once the reliability of the model has been validated with TI, which is generally done in absence of temperature effects to limit the pre-screening issues described below, TD can be applied to obtain fully converged band-shapes, in presence of temperature effects, and the details on the contribution of the most intense transitions superimposed to the result. For the sum-over-states approach, the class-based pre-screening method described in Santoro et al. (2007a,b, 2008), Barone et al. (2009), and Bloino et al. (2010) has been applied to select *a priori* the most intense transitions within a virtually infinite set. We refer interested readers to those articles for theoretical details on the algorithm. We will just mention here that the pre-screening relies on an internal database built from the transition intensities of overtones (vibrational progressions) and 2-modes combinations (mode couplings) to set the highest number of quanta each mode can reach when involved in 3-modes combinations (class 3) and above, up to a limit of 7 simultaneously excited modes (class 7). For class 1 (*C*_1_, overtones) and class 2 (*C*_2_, binary combinations), a maximum number of quanta reachable by each mode is set, respectively $C_{1}^{\max}=100$ and $C_{2}^{\max}=80$. For each class above, the maximum number of quanta for each mode was set so that no more that 4 × 10^8^ transitions are treated. For the path integral formalism, the autocorrelation function was computed over a total time of 10^−10^ s divided in 2^18^ steps (Baiardi et al., 2013). To match the experimental conditions in Sánchez-Carnerero et al. (2014), a temperature of 298 K was set.

The harmonic description used to represent the potential energy surfaces (PESs) can have a significant impact on the reliability of the overlap integrals between the initial and final vibronic states. If the minima of the PESs are nearly superimposed (small shift), the standard calculation of the force constants (Hessian matrices) at the corresponding equilibrium geometry is a good approximation of their curvatures in the region of maximum overlap. As the shift increases, this can become unsatisfactory and a better alternative is to compute the force constants in the final state about the equilibrium geometry of the initial one. The former approach will be called adiabatic Hessian (AH), and the latter vertical Hessian (VH). It should be mentioned that the vibrational energies, hence the band positions, are generally more accurate with AH, while VH can reproduce better the band intensities. However, the latter is also more sensitive to the anharmonicity of the final-state PES, with the risk of imaginary frequencies being present. Because two frequency calculations are required, which can be expensive, especially for the excited states where analytic frequency calculations may not be available, approximated alternatives have been proposed in the literature, respectively adiabatic shift (AS) for AH and vertical gradient (VG, also called linear coupling model) for VH (Blazej and Peticolas, 1980; Macak et al., 2000; Bloino et al., 2010). In those models, the final-state PES is assumed equal to the initial one. It is noteworthy that these models were primarily defined for absorption spectra, where the lower initial state is almost systematically the electronic ground state and thus less expensive to compute. This is not the case for emission spectra (OPE and CPL here), and two strategies can be adopted, depending if the state of reference, for which the frequencies are actually calculated, is the lower one, labeled with the subscript “abs” since it assumes a behavior close to absorption processes, or the excited, actual initial state, labeled with the “emi” subscript. The advantages in terms of overall computational cost of the latter is generally not considerable but will provide here a more complete picture of the validity of such approximations. Regarding the modeling of the solvent effects, for VG and VH, like for pure electronic transitions, the linear-response (LR-PCM/TD-DFT) approach for the non-equilibrium regime was employed, while the state-specific (SS-PCM/TD-DFT) was chosen for AH and AS in excited-states calculations.

Finally, both the Franck-Condon approximation (FC) and its Herzberg-Teller (HT) extension will be used for the representation of the electronic transition moments of the properties of interest, the electric and magnetic dipoles. As a matter of fact, those quantities cannot be expressed analytically with respect to the nuclear motions. The simplest way is to assume them constant, which corresponds to the FC approximation. This has two significant limitations. First, it is not able to reproduce sign alternations in the vibronic structure of chiral spectra, the spectra obtained this way being scaled versions of their non-chiral counterparts. Second, it can be a crude approximation for semi-rigid systems who undergo some deformation upon the electronic transition. A linear dependence with respect to the normal coordinates will also be included in what will be labeled the FCHT approximation. In this definition, the transition moment of a property **P** will be assumed to have the form,

$$\begin{array}{l} \text{P}\left(\text{Q}\right)=\text{P}\left({\text{Q}}^{\text{eq}}\right)+\overset{N}{\underset{i=1}{\sum }}\frac{\partial \text{P}}{\partial Q_{i}}Q_{i} \end{array}$$

where **Q** is a vector of mass-weighted normal coordinates, and **Q**^eq^ refers to the equilibrium geometry of the state of reference. The first term in the right-hand side corresponds to the FC approximation mentioned before.

In the following, the model used for the simulation of vibronic spectra will be defined starting from the framework (TI, TD), followed by the representation of the PESs (AH, VH, AS, VG), terminated by the form of the transition moments (FC, FCHT), e.g., “TI AH|FC.” Where obvious, some of the terms will be omitted for the sake of readability.

### 2.4. Internal Coordinates

A final remark concerns the choice of coordinates for the simulation of vibronic spectra. The Cartesian-based normal coordinates are often preferred for their simplicity and versatility, but can overestimate the mode mixing and the contributions of the combination bands, resulting in excessively broad bands. Internal coordinates can reduce this phenomenon, providing a better localization of the vibrational modes (Reimers, 2001; Beenken and Lischka, 2005; Borrelli and Peluso, 2008; Cerezo et al., 2013; Baiardi et al., 2016). Unfortunately, their definition is not unique and building a preliminary set containing all relevant coordinates on such a large system is not straightforward. Here, the generalized internal coordinates (GICs) implemented in Gaussian have been used. An initial set was automatically generated by the program and used as a starting point. To maximize the convergence of the spectra, *ad hoc* coordinates were defined to describe better the structural shift. Because the latter is extrapolated in the case of vertical models, different coordinates were added for adiabatic and vertical models. From them, a set of non-redundant, weighted internal coordinates (WICs, Lindh et al., 1999) was built upon it. This scheme emphasizes the localization of coordinates of different types, by weighing each one by the bond order of the atoms involved in it (Swart and Bickelhaupt, 2006). Details on the construction of the set used in our program can be found in Baiardi et al. (2016).

### 2.5. Reduced Dimensionality

For systems of this size exhibiting some flexibility, low-energy large-amplitude vibrational motions are often present and may cause a breakdown of the vibronic models despite contributing marginally to the overall spectrum. It has been shown that these modes can be ignored to provide results in very good agreement with experiment (Biczysko et al., 2011; Egidi et al., 2014, 2018; Aranda et al., 2018; Fortino et al., 2019). However, for the latter to be meaningful, it is necessary to rigorously isolate the modes to be removed from the rest of the system in a consistent way. This is done here by checking their overlap on the normal-modes basis of the other electronic state, using to this end the Duschinsky matrix, which relates the normal modes of the initial (**Q**_*I*_) and final (**Q**_*F*_) states in an affine transformation,

$$\begin{array}{l} {\text{Q}}_{I}=\text{J}{\text{Q}}_{F}+\text{K} \end{array}$$

with **J** the Duschinsky matrix and **K** the shift vector.

The so-called reduced-dimensionality procedure is as follows. For each mode of the initial (or final) electronic state, the elements of **J** (or **J**^−1^) along the corresponding row are squared and sorted by decreasing values. Then, all significant elements of this list are selected and the modes relative to the corresponding columns of **J** (or **J**^−1^) are selected to be removed too. This procedure carries on iteratively for both electronic states, alternating initial and final ones, until the lists of modes to remove stabilize with no new elements chosen. The corresponding *M* elements are removed, and the vibronic calculations are done over the (*N* − *M*) modes remaining in each state. For a system of this size, a perfect localization of the modes is extremely difficult and a residual mode mixing is generally observed over large regions. As a result, selecting all elements in a given row with non-negligible values would result in an excessive reduction of the system. In practice, a threshold is defined, here set to 0.7, so that only the first *m* elements of the list of squared row elements ordered by decreasing values are selected, so that their sum is greater or equal to the chosen threshold. It should be also noted that **J** in internal coordinates is rarely orthogonal, so that the total sum of the squared elements will vary for each row and column. For this reason, the elements of **J** and its inverse are first normalized by setting the highest element in absolute value to 1. Further details can be found in Bloino et al. (2016). As a final remark, the number of normal modes to be removed should be kept as low as possible so the model retains most of the properties of the full system.

## 3. Results and Discussions

In what follows, the results for the BODIPY dye will be presented, following the workflow described in the previous section, which can be summarized as: (i) exploration of the conformational space in order to define the low-lying conformational ensemble; (ii) choice of the best level of theory to reproduce the spectroscopic property of interest; (iii) inclusion of vibrational effects to the pure electronic transitions.

As it will be shown, the analysis of data generated in the last two steps allows for further insight on the underlying processes.

### 3.1. Conformational Analysis

**Figure 2:**
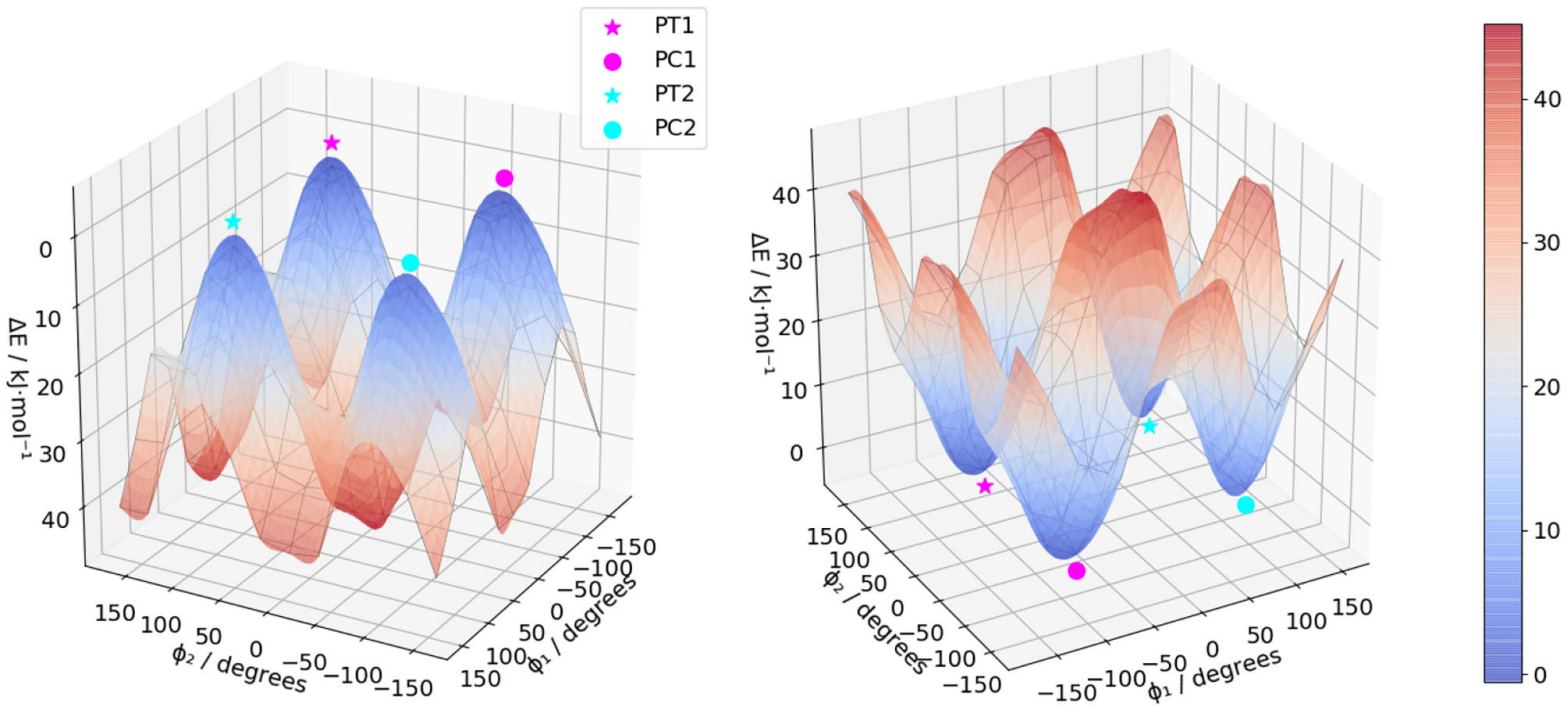
Electronic energy of (*R*)-O-BODIPY at the MN15/6-31G with different values of the torsional angles ϕ_1_ and ϕ_2_.

**Figure 3:**
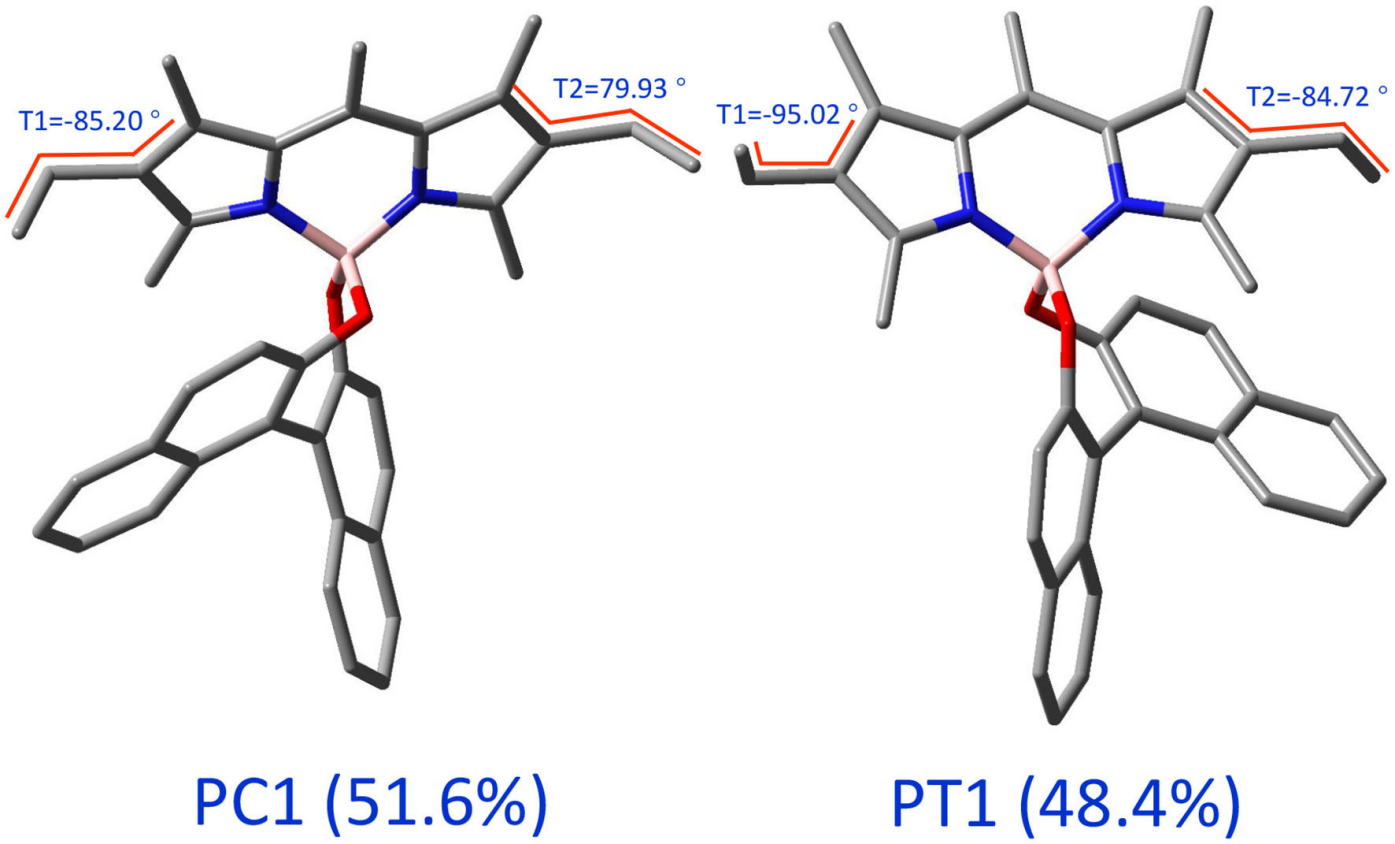
3D representations of the two types of conformers of (*R*)-O-BODIPY. The values of the angles are for the optimized geometries in the electronic ground state at the MN15/6-31+G(d).

The molecule, displayed in Figure 1, has two flexible ethyl groups, so an initial identification of the possible conformers was carried out through a relaxed scan. The values of the dihedral angles ϕ_1_ and ϕ_2_ (see Figure 1 for details) were sequentially varied from 0 to 330° by steps of 30°. The calculations were carried out in the electronic ground state at the MN15/6-31G level, this functional having shown good results on some BODIPYs systems (Fortino et al., 2019), and the overall cost being still manageable for a system of this size. As visible in Figure 2, four conformers can be identified, labeled PC1 and PC2 (for a pseudo- “cis” configuration), and PT1 and PT2 (for “trans”). Each pair of conformers is equivalent, so only one representative of each type was chosen, shown in Figure 3, simply labeled “PC” and “PT” in the following.

The overall spectra are the sum of the “PC” and “PT” spectra weighed by their relative abundances at 298 K. The free energy differences at the MN15/6-31+(d) level of theory were used to compute the Boltzmann populations at room temperature. In order to avoid non-reliable contributions due to the presence of large amplitude motions (see subsection 3.4), the vibrational correction to the free energy was neglected.

### 3.2. Electronic Structure Calculation Methods and Optical Spectra

**Figure 4:**
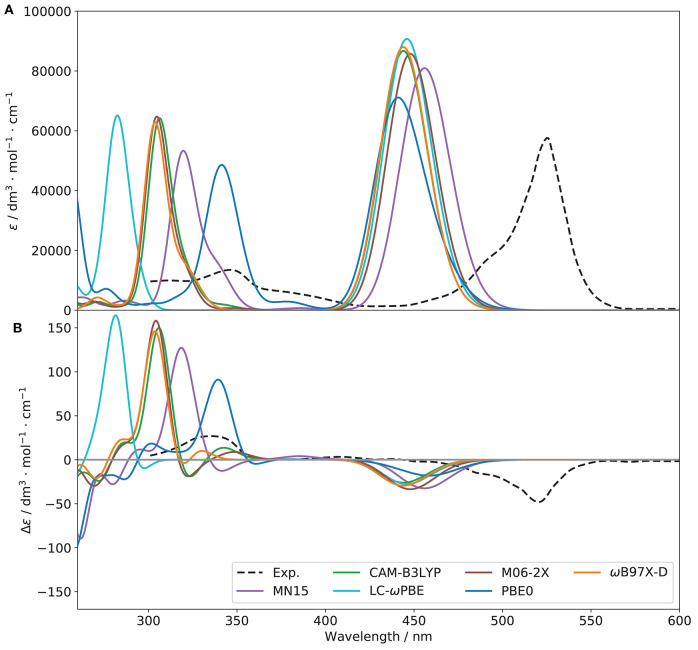
OPA **(A)** and ECD **(B)** spectra of (*R*)-O-BODIPY with different functionals [basis set: 6-31+G(d)]. The broadening was simulated by means of Gaussian functions with half-widths at half-maximum of 800 cm^−1^.

Although benchmarks on related molecules are present in literature (see for instance Fortino et al., 2019), they predominantly focus on non-chiral spectroscopies. Therefore, in order to identify the functional most suited to simulate all spectroscopies of interest, preliminary benchmarks based on both the OPA and ECD spectra were performed. A subset of functionals known to provide reliable results on similar system was chosen, namely CAM-B3LYP (Yanai et al., 2004), LC-ωPBE (Vydrov and Scuseria, 2006), M06-2X (Valero et al., 2008), MN15 (Haoyu et al., 2016), PBE0 (Adamo and Barone, 1999), and ωB97X-D (Chai and Head-Gordon, 2008). For CAM-B3LYP and PBE0, the D3 formulation of the empirical dispersion correction proposed by Grimme et al. (2010), in conjunction with the Becke-Johnson (BJ) damping (Grimme et al., 2011) were used. It should be noted that, for consistency and to avoid errors in the input files, the same parameters were used for each electronic structure calculation method, so the empirical dispersion was included in all computations involving CAM-B3LYP and PBE0. The resulting spectra are compared to experiment in Figure 4.

Considering one-photon absorption (OPA) first, all functionals give pretty similar results for the first band, shifted by about 60–70 nm with respect to experiment. The position of the second main band observed at about 350 nm is also an important indication of the relative energy gap between electronic transitions. PBE0 correctly reproduces the absolute position of the second band but not its relative energy with respect to the first one. LC-ωPBE gives the best overall result in this case, with a gap very close to the experimental one, and a spectrum shifted by about 65 nm. Regarding the shape of the bands, all functionals show a relatively similar trend in comparison with experiment, with a relatively good agreement in terms of intensity for the first band, and a significant overestimation of the second one. A low-intensity, large shoulder in the lower-energy side of the second band can be observed in the experimental spectrum, which seems to be qualitatively reproduced by PBE0. However, in terms of relative intensity with the second band, it appears underestimated, while MN15 and ωB97X-D show comparatively more intense, but narrower shoulders. Nevertheless, the limited resolution does not allow us to draw further conclusions. To conclude, LC-ωPBE provides the best relative band positions, while MN15 provides overall satisfactory band patterns, displaying the best agreement with experiment. A benchmark purely on OPA does not guarantee success in the simulation of chiroptical spectroscopies, and the performance of the functionals in describing the electronic transition moment of the magnetic dipole and its relative orientation with the electric dipole's needs to be careful evaluated too. In Figure 4B, the benchmark results for electronic circular dichroism (ECD) are reported. The ECD spectrum, like OPA, shows two main bands, of opposite sign but close height. Besides the problem of band positions, already addressed for OPA, we can note that the sign pattern is globally correctly reproduced by all functionals regarding the main features. Relatively similar heights are obtained for the first band, slightly underestimated in all cases, except for PBE0, which appears broader and weaker, while all others have only one transition in this region. The pattern for the second band is far more diverse than for OPA. All functionals significantly overestimate its intensity, and most of them also show a weaker, lower-energy band of positive (for CAM-B3LYP, ω97X-D) or negative (LC-ωPBE, M06-2X, MN15) sign, which cannot be confirmed on the experimental spectrum. The latter, however, shows a broad, nearly flat positive band at about 410 nm, which can be identified only for MN15. While clearly visible by comparison with the ECD spectrum of its enantiomer, the features of this band are too small for further analysis. Overall, the similar performance of the chosen functionals for the first transition is mostly confirmed in Table 1, except for PBE0, which exhibit two close transitions in the energy region of the first band (at about 460 and 440 nm), which complement each other for OPA but lead to a broad feature for ECD. To summarize, all functionals are capable of reproducing qualitatively the first band for both OPA and ECD spectroscopies. However OPA intensities result slightly overestimated whereas the ECD ones are underestimated. This leads to smaller values of *g*_abs_ for all the functionals compared to the experimental one, from about one fourth for PBE0 to nearly half of the value for MN15 (see Table 1 for details).

**Table 1:** Energy (*E* in nm), dipole strength (*D*, in 10^−36^ esu^2^ cm^2^), oscillator strength (*f*), and rotatory strength (*R*, in 10^−40^ erg esu cm/Gauss) for the *S*_1_ ← *S*_0_ transition.

**Functionals**	**E**	**D**	**f**	**R**	**ϵ_max_**	**Δϵ_max_**	***g*_abs_**	**Color**
MN15	456.37	58.7279	0.6001	−57.7876	80,944	−33	−4.0 × 10^−4^	
CAM-B3LYP-D3BJ	444.43	56.6468	0.6181	−45.3828	86,703	−26	−3.0 × 10^−4^	
LC-ωPBE	446.41	59.5265	0.6467	−48.0030	90,733	−28	−3.0 × 10^−4^	
M06-2X	448.39	61.1846	0.6359	−58.8453	85,734	−34	−3.9 × 10^−4^	
PBE0-D3BJ	460.81	10.0846	0.1054	−29.9259	71,107	−18	−2.5 × 10^−4^	
ωB97X-D	444.37	57.6935	0.6285	−51.1510	88,015	−29	−3.3 × 10^−4^	
Exp.	525	—	—	—	57,618	−48	−8.4 × 10^−4^	

*ϵ_max_ and Δϵ_max_ corresponds to the maximum of absorption (in OPA and ECD) for the first band (with a broadening obtained by applying Gaussian distribution functions with half-widths at half-maximum of 500 cm^−1^), and the dissymmetry ratio is computed as g_abs_ = Δϵ_max_/ϵ_max_. The perceived color is computed over the full visible spectral range*.

As MN15 shows a more consistent behavior with respect to experimental OPA and ECD spectra, and following the strategy described previously (Bloino et al., 2016), all calculations will be done from now on at the MN15/6-31+G(d). The electronic energies, and thus the band positions, will be corrected by using those obtained at the LC-ωPBE/6-31+G(d) when multiple electronic states are involved (see Figures S1, S2).

**Figure 5:**
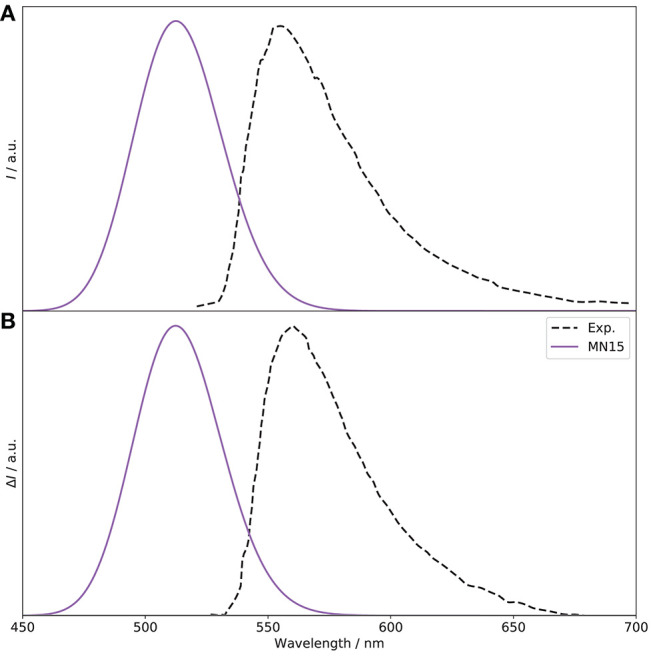
*S*_1_ → *S*_0_ OPE **(A)** and CPL **(B)** spectra of (*R*)-O-BODIPY at the MN15/6-31+G(d) level, compared to experiment. The broadening was simulated by means of Gaussian functions with half-widths at half-maximum of 800 cm^−1^. The spectra are normalized in intensity for comparison.

Moving to the emission spectra, as recalled in the introduction, the CPL and ECD experimental spectra show different signs. This is interesting because, within the Frank-Condon regime, usually the first absorption and emission transitions involve the same states, and therefore should have the same sign. In Gartzia-Rivero et al. (2017), the authors suggested that an intramolecular charge transfer (ICT) between close electronic states was likely at the origin of the change of sign observed in the experimental CPL spectrum (see Figure 5) compared to the ECD spectrum reported in Figure 4. For this reason, the first two electronic states (*S*_1_ and *S*_2_) have been considered here, and separate geometry optimizations were carried out. As expected, the sign of the CPL spectrum from the original *S*_1_ state is negative. Looking at the rotatory strengths (*R*, in the following given as 10^−40^ esu^2^cm^2^), they are respectively −34.4 for PC and −3.8 for PT, compared to −77.1 and −37.2, respectively, for the *S*_1_ ← *S*_0_ transition. Interestingly, a crossing is observed between the first and second excited singlet electronic states, with an inversion of the energy order. The previously second excited state becomes the lowest and most likely source of emission. More details on the non-radiative process requires *ad hoc* tools going beyond the scope of the present study and will be deferred to a separate work. Compared to the transition at the previous *S*_1_ minimum, PT is the largest contributor, with a rotatory strength of +30.9 (−1.9 for PC). The resulting fluorescence and CPL spectra are shown in Figure 5. A shift of about 50 nm is observed with respect to experiment, a value relatively close to absorption (≃ 60 nm), and the sign of the CPL band is correctly reproduced, with a *g*_lum_ of 0.9 × 10^−4^, compared to 7.1 × 10^−4^ reported experimentally. To get further insight, it is interesting to consider the molecular orbitals and electronic structure.

### 3.3. Molecular Orbitals and Transition Current Density

**Figure 6:**
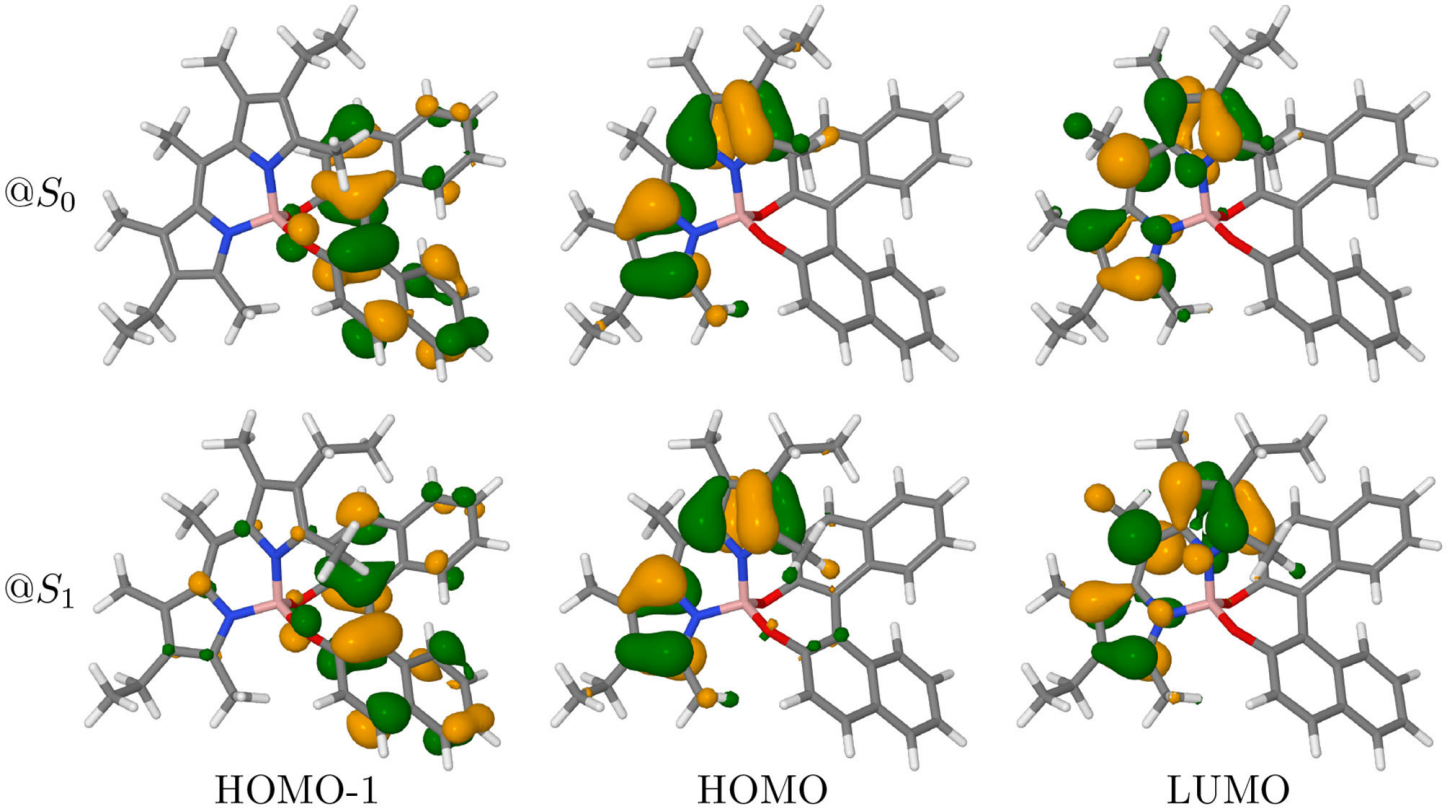
Frontiers molecular orbitals of PT conformer at *S*_0_ and *S*_1_ geometries (isodensity surfaces at ± 0.04 (*e*/*bohr*^3^)^1/2^).

**Figure 7:**
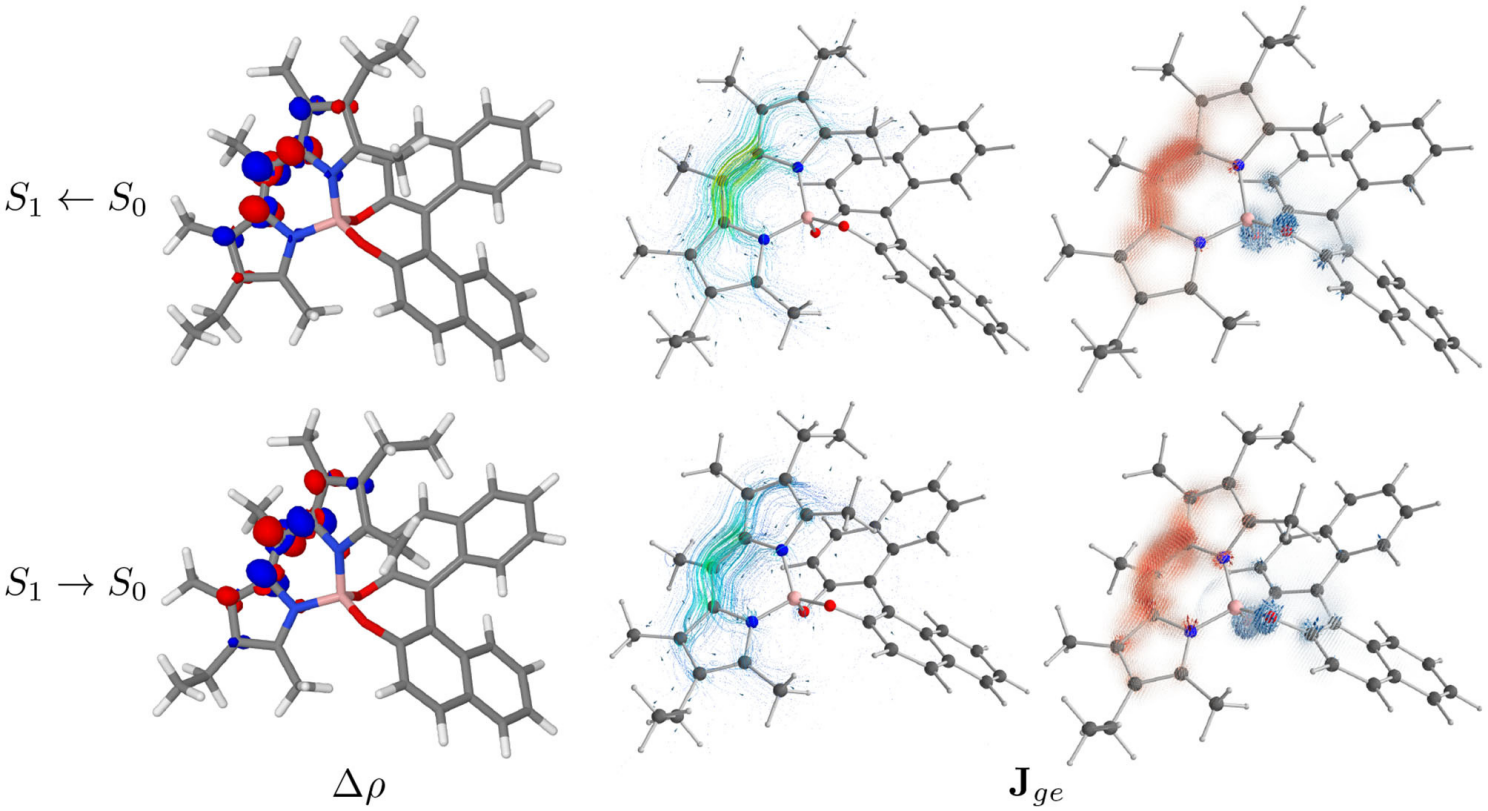
In the left panels the difference between the electronic density of the final and the initial states (Δρ) of PT conformer is represented as isosurfaces (isodensity surfaces at ± 0.004 (*e*/*bohr*^3^); red isosurfaces: depletion, blue isosurfaces: accumulation). In the central panels the ETCD (**J**_*ge*_) vector fields are represented by means of streamline objects. In the right panels, the ETCDs have been partitioned between BODIPY and BINOL contributions, with the BINOL field being magnified 15 times. The resulting fields are represented by the means of Hedgehog representation.

In the following, only PT will be discussed, as both conformers show very similar trends (the results for PC can be found in Figures S3, S4). In BODIPY systems, the lowest-energy transition is expected to be a dipole-allowed π → π^*^ transition (Bergström et al., 2002). Thus, the first absorption band is mostly described as a HOMO to LUMO transition, which contributes for more than 97% in both isomers (see Table S2). Indeed, as displayed in Figure 6, HOMO and LUMO are well-localized on the BODIPY moiety of the molecule. The figure also displays the frontier orbitals of the molecule at both the ground-state geometry and at the geometry of the lowest excited state after crossing. Though extremely similar, the HOMO and HOMO-1 are less localized at the excited state geometry. Although the lower transition still has prevalently a HOMO to LUMO nature (95%), it also has a minor contribution from the HOMO-1 to LUMO pair (4%). The electron density difference (Δρ) shown in the left panels of Figure 7 confirms the origin of the orientation of the electric dipole transition moment, but is little informative about the change in sign observed between ECD and CPL.

To investigate further the phenomenon, we look at the electronic transition current density (ETCD) (Nafie, 1994, 1997). ETCD is a vector field, which represents the paths connecting two different probability densities and its integral is associated with the velocity form of the electric dipole transition moments. What makes appealing this density function, as firstly described by Nafie and co-workers (Freedman et al., 1997, 1998, 2002; Nafie, 1997) and recently re-proposed for the vibrational case (Fusè et al., 2019), is the visualization of the vector fields. It shows linear and curved patterns in the charge flow occurring upon excitation, which can be related to the electric and magnetic dipole transition moments, respectively. In the central panels of Figure 7 (Figure S4 for PC), ETCDs at the two geometry are represented using “streamtubes” representation, obtained by interpolating the vector field (Telea, 2007). The radius and the color of the represented tubes are proportional to the magnitude of the field (from low intense in light blue to high in tones of yellow). As expected in π → π^*^ transitions (Nafie, 1994), the ETCD crosses the conjugated systems with an intense linear pattern responsible of the electric dipole transition moment, coherently with the Δρ plots. Considering its *C*_2*v*_ point group symmetry, BODIPY is not providing a chiral response itself. Once a chiral group is attached, a chiral response can rise from the dipolar coupling of the electric dipole transition moments lying on the achiral chromophore (BODIPY) and the magnetic dipole transition moment originating from the chiral portion (BINOL) (Lu et al., 2016). Nevertheless, in dipole-allowed transitions, the electric dipole transition moments is expected to be orders of magnitude more intense than the magnetic ones, making difficult to spot the contributions on BINOL. Thus, we tried to partition the ETCD on the two molecular fragments, namely the BODIPY and the BINOL units. To accomplish this, we relied on the quantum theory of atoms in molecules (Bader, 1991) to partition the total space, according to the topology of the initial state electron density, into atomic basins. Then, these basins were combined according to fragments, giving the fragments' subspace. In the right panels of Figure 7, “Hedgehog” representations of the ETCD in which the BINOL contribution (depicted in blue shades) was magnified by 15 times, are reported. As can be seen, asides from the linear flow on the BODIPY fragment (in red shades), regions of circulation of charge are present on the BINOL fragment, in particular on the oxygen atoms. Tables S3, S5 report the fragments contribution to the transition dipoles and, in turn, to the dipole strength (DS) and to the rotatory strength (RS). Focusing on the *S*_1_ ← *S*_0_ transition, the major contribution determining the sign of the first ECD band is the interaction term between the electric dipole transition moment on the BODIPY and the magnetic one on the BINOL. By comparing the upper and the lower panels in Figure 7, clues on the origin of the sign inversion between ECD and CPL can be obtained. In fact, the bending of the BODIPY frame upon excitation (see Figure S20 and *vide infra* for a comparison between the two structures) leads to a slight reduction of the linear flow and to more curved flows in portions of the BODIPY itself. As a result, the interaction term between the electric dipole transition moment on the BODIPY and the magnetic one on BINOL is reduced whereas the BODIPY intra-fragment term and the coupling between the magnetic dipole transition moment on the BODIPY and the electric one of the BINOL gains more relevance. These rearrangements in the flows in the *S*_1_ → *S*_0_ transition substantially revert the sign of CPL in the PT case and almost cause the cancellation of the CPL signal in the PC one (changes in the ETCD vector fields between transition from the first two excited states at their respective minima are shown in Figure S5).

### 3.4. Vibrationally-Resolved Electronic Spectra

The electronic structure provides already interesting information in understanding the experimental results. However, they neglect the vibrational structure contained in the experimental spectra, which can play a significant role in chiroptical spectroscopies (Lin et al., 2008a,b, 2009; Bloino et al., 2010; Pritchard and Autschbach, 2010; Santoro and Barone, 2010; Egidi et al., 2013, 2018).

To get a more accurate and complete picture, a proper account of the former is necessary. As shown in the previous section, a relatively broad range of models can be built, and it is useful to correctly assess their strengths and limits in light of the present system. Because it offers a balanced representation of the two PESs involved in the electronic transition, AH is first used as reference on the OPA and ECD spectra to check the basic setup in the definition of the vibronic system, namely the underlying coordinates system and the applicability of reduced-dimensionality schemes.

#### 3.4.1. Coordinate Systems

**Figure 8:**
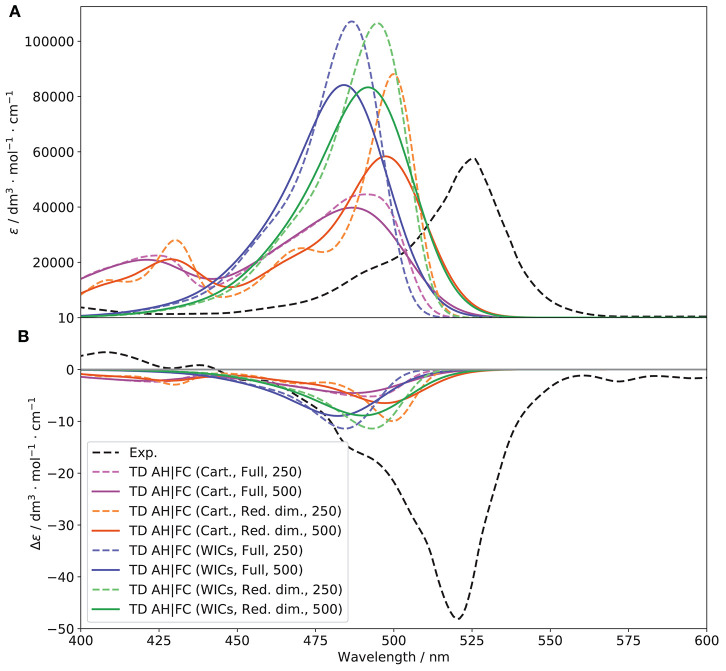
Vibronic *S*_1_ ← *S*_0_ OPA **(A)** and ECD **(B)** spectra of (*R*)-O-BODIPY at the AH|FC level within the time-dependent framework at *T* = 298 K, compared to experiment. The broadening was simulated by means of Gaussian functions with half-widths at half-maximum of 250 (dashed lines) and 500 cm^−1^ (solid lines).

Let us initiate with the coordinates. As noted before, Cartesian coordinates provide an unequivocal basis, easier to manipulate. However, it can perform poorly in presence of a system undergoing structural changes associated to the electronic transition by overestimating the mode mixing induced by it. Conversely, curvilinear linear coordinates can do a better job in describing the transformation, localizing the coordinates involved and recovering some of the intrinsic anharmonicity of the vibrational motions, reducing this way the coupling between the modes and resulting in narrower bands. However, this can be achieved only with a suitable set of coordinates, able to represent in a minimal basis the structural changes. For AH, the initial, automatically generated set of GICs, combined with the use of weights to build the non-redundant group of internal coordinates was sufficient (Lindh et al., 1999; Baiardi et al., 2016). The *S*_1_ ← *S*_0_ OPA and ECD spectra are shown in Figure 8. The time-dependent framework was chosen to avoid convergence issues, which could distort the analysis. Only the FC approximation is considered at first, so that the electronic transition moments of the electric and magnetic dipoles act as simple scaling factors, and the band-shape is dictated purely by the vibronic structure. To visualize better the impact of vibronic transitions, two different widths of broadening were applied, with half-widths at half-maximum of 500 cm^−1^ to match more closely experiment, and 250 cm^−1^ for finer details. As expected, all models are able to recover the asymmetry of the experimental first band. The shift with experiment is also improved with an overall shift of about 30 nm. However, the OPA band with Cartesian-based normal modes (“Cart”) is excessively wide. Furthermore, it exhibits a second, relatively flat band at about 420 nm not observed in the experimental spectrum. Reducing the broadening from 500 to 250 cm^−1^ barely improves the band shape. At variance, internal coordinates (“WICs”) provide a narrower, more intense band, with a smaller shift from the experimental band by about 10 nm (482 compared to 490 nm). The second band is not present here, which confirms that this is an artifact caused by the coordinates set. A flat band, even with a lower empirical broadening, is generally due to the contributions of a multitude of low-intensity transitions, which hint at an excessive mixing of the modes. This can be confirmed by analyzing the Duschinsky matrix **J**. As a visual aid for the analysis, **J** and the shift vector are graphically represented in Figures S6, S7, respectively. A few introductory comments on the chosen representations are in order. Displaying the raw numbers would produce excessively large, truncated tables, which would be difficult to analyze. Thus, it is convenient to represent each element as a square block filled with a shade of gray corresponding to the squared value of this element (${J_{ik}}^{2}$), from white for a null value to black for 1. Because **J** is not orthogonal for internal coordinates, each row was individually normalized. The shift vector is represented in absolute value, since the most important information here is the magnitude of the shift. The pattern exhibited by **J** is relatively similar between the two sets of coordinates, with a block-diagonal structure mostly close to the diagonal, which indicates that the mode mixing is not excessive and mostly involve modes close in energy, in particular in the mid-infrared region. The magnitude of the coupling is, however, harder to assess with the normalization scheme applied, and its impact on the intensity of the combination bands can be only inferred this way. To evaluate better the effects of the magnitude of the couplings, a basic sum-over-states calculation was done with the same parameters to generate the so-called Sharp and Rosenstock **C** matrix (Sharp and Rosenstock, 1963), given as,

$$\begin{array}{l} \text{C}=2{\Gamma_{F}}^{1/2}{\left({\text{J}}^{T}\Gamma_{I}\text{J}+\Gamma_{F}\right)}^{-1}{\Gamma_{F}}^{1/2}-\text{I} \end{array}$$

with **Γ** the diagonal matrix of reduced frequencies.

**Figure 9:**
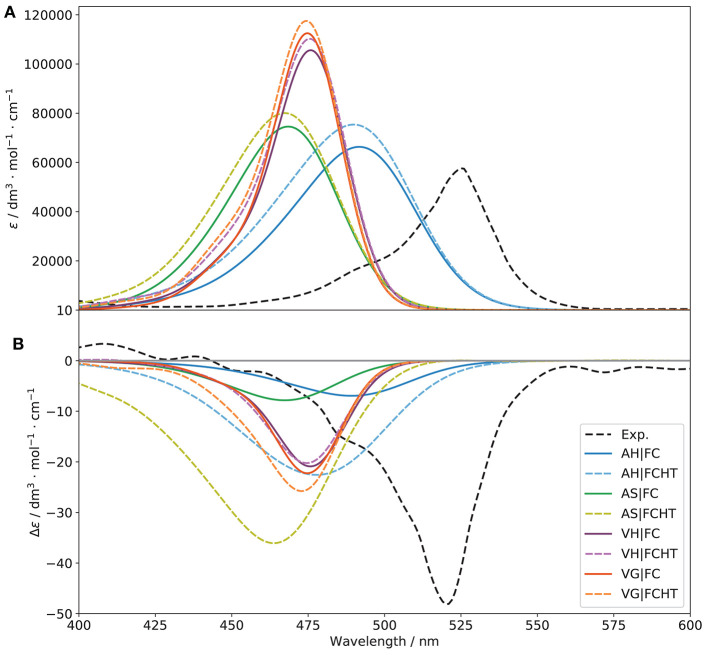
Vibronic *S*_1_ ← *S*_0_ OPA **(A)** and ECD **(B)** spectra of (*R*)-O-BODIPY computed with different vibronic models at *T* = 298 K, with and without Herzberg-Teller effects, compared to experiment. The broadening was simulated by means of Gaussian functions with half-widths at half-maximum of 500 cm^−1^.

The interest of **C** is that it is directly related to the intensity of the combination bands in the recursive formulation proposed by Ruhoff (1994) through its off-diagonal elements, and is even the only contribution in absence of temperature effects (it remains a predominant term at room temperature). The **C** matrix (shown in Figure S8) shows more difference between the coordinates sets, and in particular a large coupling of modes at higher energy (195–220) with the rest of the system, which explains the large contributions of combinations bands in Cartesian coordinates. It is noteworthy that the diagonal terms of those modes are also larger, which result in a higher contribution of the corresponding overtones in the energy region of the second band observed for this case. The shift vector (Figure S7) shows a significant shift of the first mode and in general the lower-energy ones. The combination of low energy and large shift make their contribution to the main band features marginal but will often cause an excessive broadening of the bands. For this reason, modes below 50 cm^−1^ and with shift above 100 $\sqrt{m_{e}}a_{0}$ were initially set to be removed. Following the block construction algorithm described in the computational details, five modes were removed for PC, and eight for PT. The resulting spectra are displayed as “Red. dim.” in Figure 9. We observe for both coordinates sets a narrowing of the band. However, the overall shape is still incorrect for Cartesian-based coordinates, with the second band being even more visible. This is to be expected, as low-energy modes, which contributed to some of the overall broadening are removed, while the higher-energy ones, whose vibrational progressions played a role in the second band are still present. For ECD, the results are more difficult to interpret. The intensity appears very low compared to the pure electronic one which matched quite well the first band but strongly overestimated the second, hinting at a likely compensation of errors in this case. Nevertheless, “WICs” with the reduced-dimensionality scheme seems to perform slightly better in terms of intensity. For this reason, and in the following, internal coordinates in conjunction with a reduced model system where strongly displaced, low-frequency modes are removed, will be used. As a final check, the convergence of the sum-over-states approach in the same conditions was evaluated to confirm that there was no fundamental issue with the general Franck-Condon principle used for the vibronic calculations (spectra are shown in Figure S10). Convergences of 84.7 and 92.7% were obtained for PC and PT, respectively, relatively high values in comparison of the system size and flexibility, which confirm the good performance of the overall model. It should be noted that, besides the problem of convergence highlighted earlier, the explicit sum of single vibronic transitions is computationally slow due to the manifold of initial vibrational states to take into account. For these reasons, calculations will be done within the TD framework to match experimental conditions at room temperature.

#### 3.4.2. Vibronic Models

Figure 9 shows the *S*_1_ ← *S*_0_ OPA and ECD spectra with different representations of the PES involved in the transition, with and without Herzberg-Teller effects. Normalized spectra, which emphasize the changes in the shape of the bands are shown in Figure S9.

Let us first consider the non-approximated models, AH and VH. For VH, the *S*_1_ harmonic force constants are computed at the *S*_0_ equilibrium geometry, and the shift vector, which is normally related to the structural differences between the minima of the two PESs is extrapolated assuming a parabolic curvature. As a result, the Duschinsky matrix (represented in Figure S11) and the shift vector (Figure S12) are expected to be different. As a matter of fact, **J** is slightly more diagonal, hinting at a lower mode mixing. The shift vector, however, shows one of the potential pitfalls of vertical modes, with an excessively large shift of the low-energy modes, beyond 1,000 atomic units ($\sqrt{m_{e}}a_{0}$). This is related to the definition of **K** itself, which has a magnitude roughly proportional to the squared inverse of the energy of the vibrations. For this reason, the low-frequencies can exhibit unphysical shifts. Following a common practice adopted for vertical models, modes with wavenumbers below 150 cm^−1^ were selected to be removed in the reduced-dimensionality scheme, which corresponds to about 16 modes out of 234 for both conformers. This model system has been used to generate the VH (and VG) spectra shown in Figure 9. As was inferred from the shape of **J**, VH provides a narrower band-shape compared to AH, an observation confirmed by the shape of the C matrix (Figure S13). Moreover, the high-energy wing shows a small shoulder, in agreement with experiment where it is, however, more pronounced. For ECD, the intensity is still significantly underestimated but the agreement slightly improves for VH especially in terms of band width, too large with AH. Inclusion of HT contributions improves marginally the intensity of the OPA band for VH, and has no noticeable effect on ECD. For AH, the intensity improves but the width also increases, as visible on the normalized spectra. Overall, AH tends to overestimate the mode mixing, leading to an excessive band broadening. VH appears to perform better, with a good overall band-shape, still strongly underestimated for ECD, which may be due to limitations in the ability of the underlying electronic structure calculation methods in fully describing the electronic transition magnetic dipole moment or its relative orientation with the electric dipole. The good performance can be explained by the underestimation of the structural shift from the extrapolation, which leads to a better overlap with the initial state and compensate the limitations of the harmonic approximation. At variance, the BODIPY moiety at the true *S*_1_ minimum is visibly displaced compared to *S*_0_, in particular the alkyl groups attached to it (see Figure S14 for a comparison of the reference structures used by each models). As mentioned in the computational details, the main advantage of approximated models, AS and VG, is to reduce, potentially in a significant way, the computational cost by assuming that the two PESs are equal. In practice, since the normal modes are the same in both states, there is no mode mixing (**J** is the identity matrix). Based on these observations, the results obtained here are not necessarily intuitive. Indeed, because of the lack of mode mixing, narrower, more intense bands would be expected. For VG, on OPA, the bands with FC or FCHT are very close to their VH counterparts. This can be related to the limited mode-mixing observed for VH, so VG is a good approximation for this case, and with the limited definition of the band. The same trend is observed for ECD, with a slight improvement of the band intensity with FCHT. For AS, the results are close to AH, with a slightly larger band for both OPA and ECD. Hence, the mode mixing seem to help mitigating some of the larger shifts (the shift vector is the same for AS and AH), so that AS is here poorly suited to describe such transitions.

**Figure 10:**
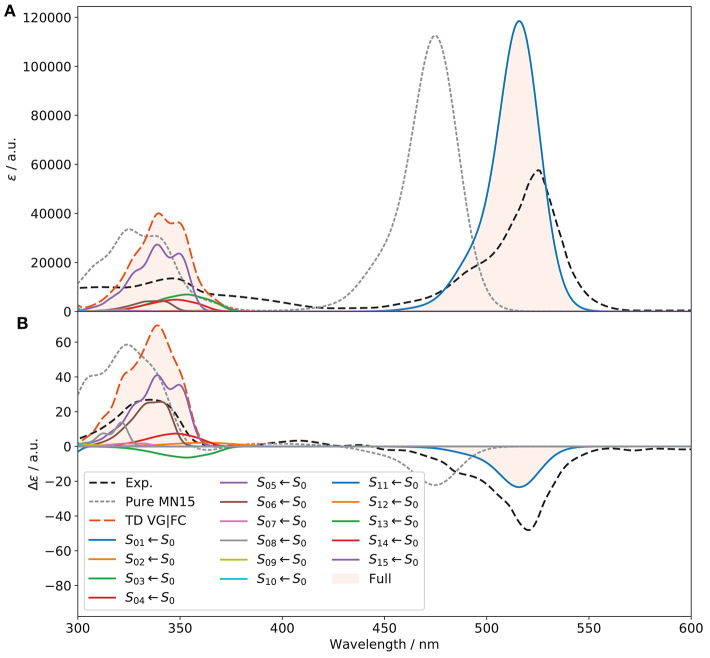
Full vibronic OPA **(A)** and ECD **(B)** spectra of (*R*)-O-BODIPY at the VG|FC level within the time-dependent framework at *T* = 298 K, compared to experiment. The electronic transitions from *S*_0_ to the first 15 electronic states are included. The broadening was simulated by means of Gaussian functions with half-width at half-maximum of 500 cm^−1^. Only the first 12 excitations are visible within the displayed spectral range.

The good performance of VG|FC makes it applicable to simulate the OPA and ECD spectra of (*R*)-O-BODIPY over a larger energy range, involving multiple electronic states. To do so, energy gradients of the first 15 excited electronic states were computed at the *S*_0_ equilibrium geometries and the relative *S*_*n*_ ← *S*_0_ OPA and ECD spectra simulated. Then, the resulting spectra were combined to obtain Figure 10. For OPA, a significant improvement of the second band experimentally at about 350 nm can be noted, but the overestimation of the first band has slightly worsened. The band positions are also shifted toward a better agreement. For ECD, the higher-energy band has improved in terms of position, shape and intensity. The low-intensity band at about 410 nm in the experimental spectrum is nearly invisible in VG|FC, similarly to what was seen for the pure electronic spectrum. In this case, inclusion of HT contributions could be beneficial, but the calculation of the derivatives of the transition dipole moments over this large number of states would significantly increase the overall computational cost. The first band, due to the *S*_1_ ← *S*_0_ transition, is still strongly underestimated, with a *g*_abs_ of about −2 × 10^−4^.

**Figure 11:**
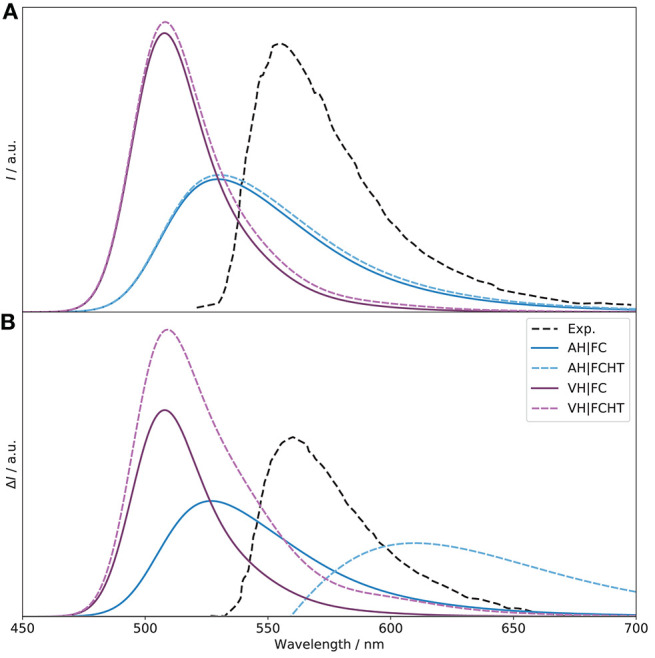
Vibronic *S*_1_ → *S*_0_ OPE **(A)** and CPL **(B)** spectra of (*R*)-O-BODIPY computed with the AH and VH models at *T* = 298 K, with and without Herzberg-Teller effects, compared to experiment. The broadening was simulated by means of Gaussian functions with half-width at half-maximum of 500 cm^−1^.

Let us now consider the emission spectra. In absence of explicit units for the experimental spectra (Sánchez-Carnerero et al., 2014), the same scale as for the electronic OPE and CPL spectra was used to facilitate our discussion. The simulated spectra obtained with the adiabatic and vertical models are shown in Figure 11 (the normalized spectra are shown in Figure S15). For OPE, the intensity of the VH|FC band is slightly higher than the pure electronic one, a trend relatively similar to what was observed for OPA. The asymmetry of the experimental shape is well-reproduced. HT effects are small, which is to be expected since the transition probability is relatively high (oscillator strength of 0.6). For AH, an excessive broadening is observed here in addition to a significant lowering of the intensity, halved. These observations mirror what was found for OPA. Looking at the Duschinsky matrix and shift vector (Figures S16, S17), we can observe more differences between AH and VH than for OPA. Indeed, the mode mixing is significantly higher, in particularly for low- and high-energy modes, and the shift vector has more elements with high amplitudes. As a matter of fact, to obtain the AH|FC spectrum shown in Figure 11, modes below 100 cm^−1^ were systematically removed, resulting in the first 30 modes excluded after consistency check (the criteria and number of modes excluded remain unchanged for VH). Even with this smaller system, the coupling remains large, as can be observed in the **C** matrix (Figure S18), which exhibits a very large number of non-negligible off-diagonal elements of magnitude similar to the diagonal terms. Looking at the geometrical changes between the *S*_1_ (noted ${}^{S_{2}}S_{1}$ since originally *S*_2_ at the *S*_0_ equilibrium) and *S*_0_ minima (Figure S19), we can note important shifts of the BODIPY moiety, with an overall tilt, combined with rotations of the peripheral alkyl group. As the electronic transition is mainly centered on this fragment of the molecule, the overall shift explains primarily the poorer performance of AH. When comparing the equilibrium geometries of the ground and first two excited electronic states (Figure S20), we can observe that the changes related to the absorption process are relatively contained, while the starting geometry for the emission process is further shifted, which explains the worse performance of the adiabatic model. Conversely, the calculated shift from VH is relatively small, leading to an extrapolated equilibrium geometry very close to the minimum of the initial state, *S*_1_ (${}^{S_{2}}S_{1}$ in the figure). Like VH, HT contributions for AH are marginal. Shifting to CPL, VH|FC reproduces well the fitted experimental spectrum, with an intensity higher than the pure electronic one. The sign is also correctly predicted. The HT effects are here apparent, leading to an increase of the intensity by about 40%, bringing the VH|FCHT intensity close to the electronic one, but with a stronger luminescence dissymmetry ratio *g*_lum_ of 1.5 × 10^−4^ (1.0 × 10^−4^ for VH|FC) and a good qualitative agreement with the experimental band-shape. Since electronic transition dipole moments act as scaling factors within the FC approximation, the excessive broadening of AH|FC observed for OPE is visible on CPL too. The intensity remains low compared to VH|FC too. Adding HT contributions results in a sign alternation with a first negative band at about 540 nm followed by a wide positive band above 600 nm (see Figure S21 for details). This phenomenon is due to the fact that at this level, the two conformers have broad band-shapes of opposite signs but similar magnitudes, resulting in a destructive combination. The sign alternation reflects the dominance of one conformer's contribution over the one, with PT negative and PC positive.

Let us conclude our analysis on the use of approximated models. As explained in the computational details, their definition is less straightforward than in absorption. Indeed, from a theoretical point of view, it is assumed that the PES remains unaltered by the transition, simply shifted, so that the PES of the final state is the same as the initial one. From a computational perspective, excited-state frequency calculations are generally more expensive, in particular in absence of analytical frequencies. For emission spectra, theoretical and practical considerations have opposite implications. The two are shown in the Supplementary Material (Figures S21, S22 for the normalized spectrum), “abs” using the ground state as reference, “emi” the initial, exited state. Both approximated vertical models give very close results for OPE and HT contributions are negligible. On the other hand, adiabatic models perform poorly, with AS_abs_ giving a nearly flat spectrum, with a peak at about 200 nm. This behavior can be directly related to the significant structural shift between the minima of the two states, mitigated by the mode mixing in AH. Switching to a description based on the normal coordinates of the excited state (AS_emi_) improves the results, but with still an excessive mixing. As for OPA, HT contributions are very small. On CPL, the trend remains relatively similar for all methods at the FC level. However, inclusion of HT effects give very disparate results. While VG_abs_ produces a significantly narrower and more intense band compared to VH with a slightly negative, flat band between 550 and 650 nm, VG_emi_ exhibits an almost symmetric pattern, starting with a relatively narrow negative band, followed by a low-intensity positive band. Both diverge significantly from VH and the experimental data. AS_abs_ remains unusable at all levels, and AS_emi_ gives the same, broad and weaker band with and without HT contributions. These results show that approximated modes need to be carefully handled and can give poor results if the structural deformations are non-negligible.

### 3.5. Influence of the Vibronic Structure

Based on its performance, VH is used here for the analysis of the band contributions. TI VH|FC and VH|FCHT calculations were done in the same conditions as TD, with different broadenings to see the evolution of the band pattern. In the following, the transitions of interest are reported with their intensity given in terms of rotatory strength (*R*, in 10^−44^ esu^2^cm^2^).

Starting from the ECD spectra (shown in Figure S23), the 0–0 transition, between the vibrational ground states, is the main transition at both FC and FCHT levels for both PC (*cis*-conformer) and PT (*trans*-conformer). The intensity of the main transition of PC is three times that of PT at 477 nm in the spectra (−114.545 vs. −40.009). Hence, increasing the abundance of the *cis* conformer through structural modifications or environmental perturbations will lead to a higher ECD signal. The position of the band maximum is modulated by the contributions of lower-intensity transitions. For the PC conformer, fundamentals of modes 22, 62, and 137, at 473, 465, and 451 nm, respectively, are visible at both FC and FCHT levels. These three modes correspond to vibrations centered on the BINOL moiety, especially for mode 137, fully localized on the BINOL part. Mode 22 is also accompanied by a motion of the ethyl groups, while mode 62 also involves a ring distortion on the achiral perylene moiety. For PT, the 0–0 transition is clearly dominant at both FC and FCHT level. The other transitions have a small impact at the FC level, while contributions from the fundamentals of modes 20, 22, and 50, at 474, 473, and 468 nm, respectively, can be noted with FCHT. Similarly to PC, these three modes are strongly connected to the BINOL moiety, especially vibrational mode 50, characterized by a bending of two naphthol groups. For modes 20 and 22, motions of the methyl or ethyl groups can also be observed. From the vibronic analysis above, it can be concluded that the vibrational motions of the BINOL moiety give rise to the most significant vibronic transitions. This is also in line with the analysis from ETCD and the structural differences reported in Figure S14. Besides the relative weights of PC and PT, the contribution of modes related to BINOL motions is also another important entry point for tuning the ECD properties of this system, even if the relatively sparse vibronic contribution makes a tailored modification of the current structure difficult to anticipate.

**Figure 12:**
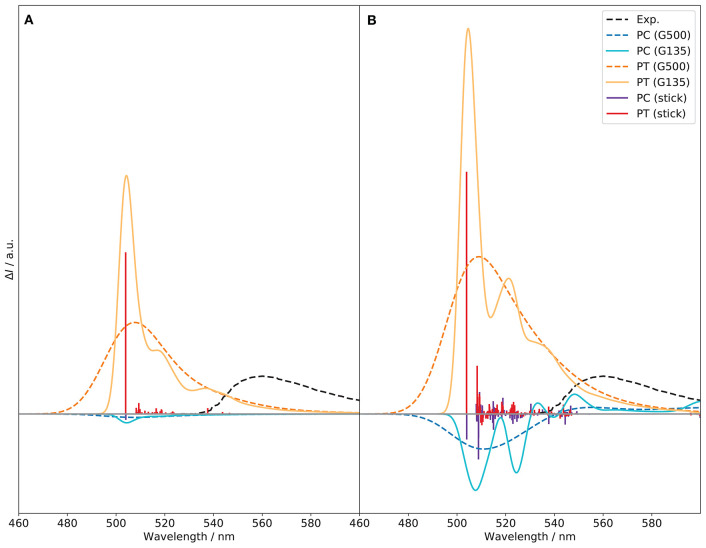
Vibronic *S*_1_ → *S*_0_ CPL spectra of the PC and PT conformers of (*R*)-O-BODIPY computed at the TI VH|FC **(A)** and VH|FCHT **(B)** levels with different broadenings. Gaussian distribution functions are used with half-widths at half-maximum of 135 (“G135”) and 500 cm^−1^ (“G500”). The stick spectra were arbitrarily scaled to fit in the figure.

The vibronic structure in the CPL spectra, shown in Figure 12, is richer than for ECD. At variance with the ECD spectrum, the PT conformer is the largest contributor and gives the sign of the CPL band, both at FC and FCHT levels, whereas PC, of opposite sign, has a canceling effect. Therefore, reducing the population of the *cis*-conformer for this system would lead to a notable enhancement of the CPL signal. At the FC level, the transition at about 504 nm, between the vibrational ground states, dominates for both PC and PT, but with opposite signs. The rotatory strength of the 0–0 transition of PC is negative and about thirty times lower in magnitude than PT (−2.084 vs. 56.974). Then, the most notable contribution is the fundamental of mode 25 of PT at 509 nm, with an intensity doubled compared to the 0–0 transition of PC. This mode is characterized by the bending of the two naphthol groups in the BINOL moiety, accompanied by a slight motion of the ethyl groups. This vibration matches the displacement of the naphthyl group observed between the *S*_1_ and *S*_0_ true minima reported in Figure S19, which is in line with the vibrational progression observed. Aside from this one, modes 53 and 138, at 516 and 551 nm, respectively, also contribute noticeably to the CPL spectrum of PT. Mode 53 is characterized by a deformation of the rings on the BINOL moiety, while mode 138 corresponds to a deformation of the six-member ring in the BODIPY unit around the boron atom. For PC, no other noticeable contributions can be identified at the FC level. At the FCHT level, an enhancement effect observed on the overall band-shape for both conformers, which can be related to a few significant changes in the vibronic transitions. The intensity of the fundamental transitions for mode 25 in PT is doubled, while modes 53 and 138 are increased by one fourth. Additional contributions become more visible, related to the fundamentals of modes 19, 20, 24, 27, 42, 61, 75, 78, and 163. Except for mode 163, relatively similar to mode 138, the other vibrations are directly related to the BINOL moiety. For PC, HT contributions give rise to new vibronic contributions. The most significant one is the fundamental transition of mode 22, which has twice the intensity of the 0–0 one (−16.622 vs. −9.010). The energy of mode 22 is very close to mode 25 of PT, at 509 nm, and corresponds to a very similar vibration, characterized by the bending of the naphthol groups in the BINOL moiety. By its major contribution, this transition causes a slight shift in the emission maximum with respect to PT. The transitions to the fundamentals of modes 23 and 25 should be also mentioned, with intensities comparable to the 0–0 transitions. Both are characterized by the bending of the two naphthol groups in the BINOL moiety but with opposite sign (23 is negative, 25 positive). The vibrational analysis of the CPL spectra shows the significant contribution of the BINOL unit to the CPL sign, shape and intensity for both PC and PT. Therefore substitutions aimed to move the center of mass toward the BINOL fragment can sensibly modify the band shape and in turn the position of the band. The strategy can be combined with *ad hoc* modifications of the push-pull property of the molecule. This is also in line with ETCD Results, that an higher electron current density within the BINOL can lead to an enhancement of the CPL signal.

## 4. Conclusions

We present here an extensive protocol for the study of chiroptical spectra of medium-to-large molecular systems beyond the standard, purely electronic, methods. The inclusion of vibrational contributions improves on overall the agreement with experiment, giving further insights on the origin of the spectral band-shape observed experimentally. Coupled with tailored graphical representations, like electronic transition current density, which shows the local contributions to the electric and magnetic transition dipole moments, it allows identifying the regions contributing more to the main features and the intensity of the bands. By identifying the excited states actually involved and the weight of each conformer in the CPL spectrum, it is possible to provide precise band assignments. This information can in turn be used to rationalize the design choice and propose paths to improvements in the performance of chiral BODIPYs, both in terms of intensity of emission and luminescence dissymmetry ratio. The results confirm the key role played by the conformational analysis in spectroscopic studies, especially in the present case. Two major conformations were found with opposite chiroptical properties in emission. The performance of the investigated functionals for the first band is close, with some marked differences at higher energies and for the chiral spectra. Overall, MN15 showed more consistent results for both OPA and ECD spectra, confirming to be a reliable choice for describing BODIPY systems (Fortino et al., 2019). Concerning vibronic effects, several aspects can be tuned to better describe the system: (i) Internal coordinates can better represent structural changes induced by the electronic transition, thus whenever a suitable set of coordinates are available they should be preferred over Cartesian ones. In the present system, weighted coordinates (WICs) prove to be particularly effective; (ii) The analysis of the Duschinsky transformation, namely the **J** matrix and **K** vector, aided the choice of the most suitable vibronic model and, combined with the **C** matrix (from time-independent calculations), the definition of the reduced model. Here, vertical models performed better, in particular the VH model resulted to be the best suited for both absorption and emission spectra, which is expected for low-resolution spectra were a correct description of the peaks intensity is more critical. In absorption spectra, the good performance of VG allowed to extend the vibronic simulation on a wider energy range. Finally, TI spectra computed with the best model can be used to highlight the most relevant vibrational contributions.

Besides the clear value in supporting experimental studies, such a comprehensive protocol is also relevant as a diagnostics tools for approximated methods, including those based on pure electronic structure calculations. Indeed, a proper account of the vibrational structure can help check the quality of the potential energy surface, and will modulate the position of the band-shape and its intensity, revealing potential risks of error compensation. Furthermore, Herzberg-Teller terms can highlight important intensity-borrowing effects, potentially causing significant shift in intensity and band patterns.

Thanks to ongoing efforts in different groups, vibronic spectra can be routinely computed for a growing number of systems of increasing size and complexity. Nevertheless, structural deformations associated to the electronic transition still represent a complex challenge to reach a full black-box procedure. The choice of coordinates can play a critical role in the quality of the band-shape, and the treatment of large amplitude motions, which often give very low contributions, remains a difficult task. Automated procedures can now significantly reduce the work required to set up a proper computational protocol, but still require some careful checks of the produced data. By exploiting some of the representations shown here, paths of improvements can be devised in making such controls autonomous, with little input requested from the users.

## Data Availability Statement

The raw data used to generate the figures and supporting the conclusions of this article are available upon request.

## Author Contributions

All authors listed have made a substantial, direct and intellectual contribution to the work, and approved it for publication.

## Conflict of Interest

The authors declare that the research was conducted in the absence of any commercial or financial relationships that could be construed as a potential conflict of interest.

## References

[B1] Abbate S. Bruhn T. Pescitelli G. Longhi G. (2017). Vibrational optical activity of BODIPY dimers: the role of magnetic–electric coupling in vibrational excitons. J. Phys. Chem. A 121, 394–400. 10.1021/acs.jpca.6b11327 27973781

[B2] Adamo C. Barone V. (1999). Toward reliable density functional methods without adjustable parameters: the PBE0 model. J. Chem. Phys. 110, 6158–6170. 10.1063/1.478522

[B3] Alnoman R. B. Rihn S. O'Connor D. C. Black F. A. Costello B. Waddell P. G.. (2016). Circularly polarized luminescence from helically chiral N,N,O,O-boron-chelated dipyrromethenes. Chem. Eur. J. 22, 93–96. 10.1002/chem.201504484 26555772 PMC4736443

[B4] Aranda D. Cerezo J. Pescitelli G. Ferrer F. J. A. Soto J. Santoro F. (2018). A computational study of the vibrationally-resolved electronic circular dichroism spectra of single-chain transoid and cisoid oligothiophenes in chiral conformations. Phys. Chem. Chem. Phys. 20, 21864–21880. 10.1039/C8CP03482F 30105334

[B5] Avila Ferrer F. J. Cerezo J. Stendardo E. Improta R. Santoro F. (2013). Insights for an accurate comparison of computational data to experimental absorption and emission spectra: beyond the vertical transition approximation. J. Chem. Theory Comput. 9, 2072–2082. 10.1021/ct301107m 26583553

[B6] Bader R. F. W. (1991). A quantum theory of molecular structure and its applications. Chem. Rev. 91, 893–928. 10.1021/cr00005a013

[B7] Baiardi A. Bloino J. Barone V. (2013). General time dependent approach to vibronic spectroscopy including Franck–Condon, Herzberg–Teller, and duschinsky effects. J. Chem. Theory Comput. 9, 4097–4115. 10.1021/ct400450k 26592403 PMC6485600

[B8] Baiardi A. Bloino J. Barone V. (2016). General formulation of vibronic spectroscopy in internal coordinates. J. Chem. Phys. 144:084114. 10.1063/1.4942165 26931688 PMC5732572

[B9] Barone V. Biczysko M. Borkowska-Panek M. Bloino J. (2014). A multifrequency virtual spectrometer for complex bio-organic systems: vibronic and environmental effects on the uv/vis spectrum of chlorophyll a. ChemPhysChem 15, 3355–3364. 10.1002/cphc.201402300 25182331

[B10] Barone V. Bloino J. Biczysko M. Santoro F. (2009). Fully integrated approach to compute vibrationally resolved optical spectra: from small molecules to macrosystems. J. Chem. Theory Comput. 5, 540–554. 10.1021/ct8004744 26610221

[B11] Beenken W. J. D. Lischka H. (2005). Spectral broadening and diffusion by torsional motion in biphenyl. J. Chem. Phys. 123:144311. 10.1063/1.2049269 16238395

[B12] Bergström F. Mikhalyov I. Hägglöf P. Wortmann R. Ny T. Johansson L. B.-Å. (2002). Dimers of dipyrrometheneboron difluoride (BODIPY) with light spectroscopic applications in chemistry and biology. J. Am. Chem. Soc. 124, 196–204. 10.1021/ja010983f 11782171

[B13] Biczysko M. Bloino J. Santoro F. Barone V. (2011). Chapter: time independent approaches to simulate electronic spectra lineshapes: from small molecules to macrosystems, in Computational Strategies for Spectroscopy, From Small Molecules to Nano Systems, ed Barone V. (Chichester: John Wiley and Sons Ltd), 361–443.

[B14] Blazej D. C. Peticolas W. L. (1980). Ultraviolet resonance Raman excitation profiles of pyrimidine nucleotides. J. Chem. Phys. 72, 3134–3142. 10.1063/1.439547

[B15] Bloino J. Baiardi A. Biczysko M. (2016). Aiming at an accurate prediction of vibrational and electronic spectra for medium-to-large molecules: an overview. Int. J. Quantum Chem. 116, 1543–1574. 10.1002/qua.25188

[B16] Bloino J. Biczysko M. Santoro F. Barone V. (2010). General approach to compute vibrationally resolved one-photon electronic spectra. J. Chem. Theory Comput. 6, 1256–1274. 10.1021/ct9006772 26610221

[B17] Borrelli R. Peluso A. (2008). The electron photodetachment spectrum of c-C_4_F_8_^−^: a test case for the computation of Franck-Condon factors of highly flexible molecules. J. Chem. Phys. 128:044303. 10.1063/1.2819061 18247945

[B18] Cancès E. Mennucci B. Tomasi J. (1997). A new integral equation formalism for the polarizable continuum model: theoretical background and applications to isotropic and anisotropic dielectrics. J. Chem. Phys. 107, 3032–3041. 10.1063/1.474659

[B19] Carr R. Evans N. H. Parker D. (2012). Lanthanide complexes as chiral probes exploiting circularly polarized luminescence. Chem. Soc. Rev. 41, 7673–7686. 10.1039/C2CS35242G 22895164

[B20] Cerezo J. Zúñiga J. Requena A. Ávila Ferrer F. J. Santoro F. (2013). Harmonic models in cartesian and internal coordinates to simulate the absorption spectra of carotenoids at finite temperatures. J. Chem. Theory Comput. 9, 4947–4958. 10.1021/ct4005849 26583413

[B21] Chai J.-D. Head-Gordon M. (2008). Long-range corrected hybrid density functionals with damped atom-atom dispersion corrections. Phys. Chem. Chem. Phys. 10, 6615–6620. 10.1039/b810189b 18989472

[B22] Durán-Sampedro G. Agarrabeitia A. R. Cerdán L. Pérez-Ojeda M. E. Costela A. García-Moreno I. (2013). Carboxylates versus fluorines: boosting the emission properties of commercial BODIPYs in liquid and solid media. Adv. Funct. Mater. 23, 4195–4205. 10.1002/adfm.201300198

[B23] Egidi F. Bloino J. Cappelli C. Barone V. (2013). Development of a virtual spectrometer for chiroptical spectroscopies: the case of nicotine. Chirality 25, 701–708. 10.1002/chir.22200 23857879 PMC4604657

[B24] Egidi F. Bloino J. Cappelli C. Barone V. (2014). A robust and effective time-independent route to the calculation of resonance raman spectra of large molecules in condensed phases with the inclusion of Duschinsky, Herzberg–Teller, anharmonic, and environmental effects. J. Chem. Theory Comput. 10, 346–363. 10.1021/ct400932e 26550003 PMC4632188

[B25] Egidi F. Fusè M. Baiardi A. Bloino J. Li X. Barone V. (2018). Computational simulation of vibrationally resolved spectra for spin-forbidden transitions. Chirality 30, 850–865. 10.1002/chir.22864 29727500 PMC6003600

[B26] Fortino M. Bloino J. Collini E. Bolzonello L. Trapani M. Faglioni F.. (2019). On the simulation of vibrationally resolved electronic spectra of medium-size molecules: the case of styryl substituted BODIPYs. Phys. Chem. Chem. Phys. 21, 3512–3526. 10.1039/C8CP02845A 30052253

[B27] Freedman T. B. Lee E. Zhao T. (2002). Vibrational transition current density: visualizing the origin of vibrational circular dichroism and infrared intensities, in Chirality: Physical Chemistry, Chapter 5, ed Hicks J. M. (Washington, DC: American Chemical Society), 65–78.

[B28] Freedman T. B. Shih M.-L. Lee E. Nafie L. A. (1997). Electron transition current density in molecules. 3. *Ab initio* calculations for vibrational transitions in ethylene and formaldehyde. J. Am. Chem. Soc. 119, 10620–10626. 10.1021/ja9701568

[B29] Freedman T. B. Shih M.-L. Lee E. Nafie L. A. (1998). Electron transition current density in molecules. 2. *Ab initio* calculations for electronic transitions in ethylene and formaldehyde. J. Phys. Chem. A 102, 3352–3357. 10.1021/jp972345i

[B30] Frisch M. J. Trucks G. W. Schlegel H. B. Scuseria G. E. Robb M. A. Cheeseman J. R. (2019). Gaussian Development Version, Revision J.05. Wallingford, CT: Gaussian, Inc.

[B31] Furumi S. (2010). Recent progress in chiral photonic band-gap liquid crystals for laser applications. Chem. Rec. 10, 394–408. 10.1002/tcr.201000013 20954194

[B32] Fusè M. Egidi F. Bloino J. (2019). Vibrational circular dichroism under the quantum magnifying glass: from the electronic flow to the spectroscopic observable. Phys. Chem. Chem. Phys. 21, 4224–4239. 10.1039/C8CP06514D 30747175

[B33] Gartzia-Rivero L. Sánchez-Carnerero E. M. Jiménez J. Bañuelos J. Moreno F. Maroto B. L.. (2017). Modulation of ict probability in bi (polyarene)-based O-BODIPYs: towards the development of low-cost bright arene-BODIPY dyads. Dalton Trans. 46, 11830–11839. 10.1039/C7DT01984J 28848944

[B34] Grimme S. Antony J. Ehrlich S. Krieg H. (2010). A consistent and accurate ab initio parametrization of density functional dispersion correction (DFT-D) for the 94 elements H-PU. J. Chem. Phys. 132:154104. 10.1063/1.3382344 20423165

[B35] Grimme S. Ehrlich S. Goerigk L. (2011). Effect of the damping function in dispersion corrected density functional theory. J. Comput. Chem. 32, 1456–1465. 10.1002/jcc.21759 21370243

[B36] Haoyu S. Y. He X. Li S. L. Truhlar D. G. (2016). MN15: a Kohn–Sham global-hybrid exchange–correlation density functional with broad accuracy for multi-reference and single-reference systems and non-covalent interactions. Chem. Sci. 7, 5032–5051. 10.1039/C6SC00705H 30155154 PMC6018516

[B37] Hodecker M. Biczysko M. Dreuw A. Barone V. (2016). Simulation of vacuum UV absorption and electronic circular dichroism spectra of methyl oxirane: the role of vibrational effects. J. Chem. Theory Comput. 12, 2820–2833. 10.1021/acs.jctc.6b00121 27159495 PMC5612404

[B38] Hu Y. Wang C.-W. Zhu C. Gu F. Lin S.-H. (2017). Franck–Condon simulation for unraveling vibronic origin in solvent enhanced absorption and fluorescence spectra of rubrene. RSC Adv. 7, 12407–12418. 10.1039/C7RA00417F

[B39] Jimènez J. Cerdàn L. Moreno F. Maroto B. L. García-Moreno I. Lunkley J. L.. (2017). Chiral organic dyes endowed with circularly polarized laser emission. J. Phys. Chem. C 121, 5287–5292. 10.1021/acs.jpcc.7b00654 28993793 PMC5630177

[B40] Jiménez J. Moreno F. Maroto B. L. Cabreros T. A. Huy A. S. Muller G.. (2019). Modulating ICT emission: a new strategy to manipulate the CPL sign in chiral emitters. Chem. Commun. 55, 1631–1634. 10.1039/C8CC09401B 30657143 PMC7063650

[B41] Kamkaew A. Lim S. H. Lee H. B. Kiew L. V. Chung L. Y. Burgess K. (2013). BODIPY dyes in photodynamic therapy. Chem. Soc. Rev. 42, 77–88. 10.1039/C2CS35216H 23014776 PMC3514588

[B42] Kaur P. Singh K. (2019). Recent advances in the application of BODIPY in bioimaging and chemosensing. J. Mater. Chem. C 7, 11361–11405. 10.1039/C9TC03719E

[B43] Kumar J. Nakashima T. Kawai T. (2015). Circularly polarized luminescence in chiral molecules and supramolecular assemblies. J. Phys. Chem. Lett. 6, 3445–3452. 10.1021/acs.jpclett.5b01452 26269090

[B44] Le Guennic B. Maury O. Jacquemin D. (2012). Aza-boron-dipyrromethene dyes: TD-DFT benchmarks, spectral analysis and design of original near-IR structures. Phys. Chem. Chem. Phys. 14, 157–164. 10.1039/C1CP22396H 22068264

[B45] Li M. Li S.-H. Zhang D. Cai M. Duan L. Fung M.-K.. (2018). Stable enantiomers displaying thermally activated delayed fluorescence: efficient OLEDs with circularly polarized electroluminescence. Angew. Chem. Int. Ed. 57, 2889–2893. 10.1002/anie.201800198 29356268

[B46] Licari D. Baiardi A. Biczysko M. Egidi F. Latouche C. Barone V. (2015). Implementation of a graphical user interface for the virtual multifrequency spectrometer: the VMS-draw tool. J. Comput. Chem. 36, 321–334. 10.1002/jcc.23785 25408126

[B47] Lifschitz A. M. Shade C. M. Spokoyny A. M. Mendez-Arroyo J. Stern C. L. Sarjeant A. A.. (2013). Boron-dipyrromethene-functionalized hemilabile ligands as “turn-on” fluorescent probes for coordination changes in weak-link approach complexes. Inorg. Chem. 52, 5484–5492. 10.1021/ic400383t 23570551

[B48] Lin N. Luo Y. Santoro F. Zhao X. Rizzo A. (2008a). Vibronically-induced change in the chiral response of molecules revealed by electronic circular dichroism. Chem. Phys. Lett. 464, 144–149. 10.1016/j.cplett.2008.09.013

[B49] Lin N. Santoro F. Rizzo A. Luo Y. Zhao X. Barone V. (2009). Theory for vibrationally resolved two-photon circular dichroism spectra. application to (*R*)-(+)-3-methylcyclopentanone. J. Phys. Chem. A 113, 4198–4207. 10.1021/jp8105925 19253990

[B50] Lin N. Santoro F. Zhao X. Rizzo A. Barone V. (2008b). Vibronically resolved electronic circular dichroism spectra of (*R*)-(+)-3-methylcyclopentanone: a theoretical study. J. Phys. Chem. A 112, 12401–12411. 10.1021/jp8064695 18998661

[B51] Lindh R. Bernhardsson A. Schütz M. (1999). Force-constant weighted redundant coordinates in molecular geometry optimizations. Chem. Phys. Lett. 303, 567–575. 10.1016/S0009-2614(99)00247-X

[B52] Liu S. Shi Z. Xu W. Yang H. Xi N. Liu X. (2014). A class of wavelength-tunable near-infrared AZA-BODIPY dyes and their application for sensing mercury ion. Dyes Pigm. 103, 145–153. 10.1016/j.dyepig.2013.12.00410.1016/j.dyepig.2013.12.004

[B53] Liu Y. Cerezo J. Mazzeo G. Lin N. Zhao X. Longhi G.. (2016). Vibronic coupling explains the different shape of electronic circular dichroism and of circularly polarized luminescence spectra of hexahelicenes. J. Chem. Theory Comput. 12, 2799–2819. 10.1021/acs.jctc.6b00109 27120334

[B54] Longhi G. Castiglioni E. Koshoubu J. Mazzeo G. Abbate S. (2016). Circularly polarized luminescence: a review of experimental and theoretical aspects. Chirality 28, 696–707. 10.1002/chir.22647 27670249

[B55] Loudet A. Burgess K. (2007). Bodipy dyes and their derivatives: syntheses and spectroscopic properties. Chem. Rev. 107, 4891–4932. 10.1021/cr078381n 17924696

[B56] Lu H. Mack J. Nyokong T. Kobayashi N. Shen Z. (2016). Optically active BODIPYs. Coord. Chem. Rev. 318, 1–15. 10.1016/j.ccr.2016.03.015

[B57] Lu T. Chen F. (2012). Multiwfn: a multifunctional wavefunction analyzer. J. Comput. Chem. 33, 580–592. 10.1002/jcc.22885 22162017

[B58] Macak P. Luo Y. Ågren H. (2000). Simulations of vibronic profiles in two-photon absorption. Chem. Phys. Lett. 330, 447–457. 10.1016/S0009-2614(00)01096-4

[B59] Nafie L. A. (1994). Molecular electronic transition current density, Chapter 4, in Molecular and Biomolecular Electronics, ed Birge R. R. (Washington, DC: American Chemical Society), 63–80.

[B60] Nafie L. A. (1997). Electron transition current density in molecules. 1. non-Born-Oppenheimer theory of vibronic and vibrational transitions. J. Phys. Chem. A 101, 7826–7833. 10.1021/jp9706137

[B61] Padula D. Santoro F. Pescitelli G. (2016). A simple dimeric model accounts for the vibronic ECD spectra of chiral polythiophenes in their aggregated states. RSC Adv. 6, 37938–37943. 10.1039/C6RA05500A

[B62] Pedone A. Bloino J. Barone V. (2012). Role of host–guest interactions in tuning the optical properties of coumarin derivatives incorporated in MCM-41: A TD-DFT investigation. J. Phys. Chem. C 116, 17807–17818. 10.1021/jp305294u

[B63] Pop F. Zigon N. Avarvari N. (2019). Main-group-based electro-and photoactive chiral materials. Chem. Rev. 119, 8435–8478. 10.1021/acs.chemrev.8b00770 30943018

[B64] Pritchard B. Autschbach J. (2010). Calculation of the vibrationally resolved, circularly polarized luminescence of d-camphorquinone and (*S, S*)-trans-β-hydrindanone. ChemPhysChem 11, 2409–2415. 10.1002/cphc.20100005410.1002/cphc.201000054 20632354

[B65] Reimers J. R. (2001). A practical method for the use of curvilinear coordinates in calculations of normal-mode-projected displacements and duschinsky rotation matrices for large molecules. J. Chem. Phys. 115, 9103–9109. 10.1063/1.1412875

[B66] Riehl J. P. Richardson F. S. (1986). Circularly polarized luminescence spectroscopy. Chem. Rev. 86, 1–16. 10.1021/cr00071a001

[B67] Ruhoff P. T. (1994). Recursion relations for multi-dimensional Franck-Condon overlap integrals. Chem. Phys. 186, 355–374. 10.1016/0301-0104(94)00173-1

[B68] Sánchez-Carnerero E. M. Agarrabeitia A. R. Moreno F. Maroto B. L. Muller G. Ortiz M. J.. (2015). Circularly polarized luminescence from simple organic molecules. Chem. Eur. J. 21, 13488–13500. 10.1002/chem.201501178 26136234 PMC4567477

[B69] Sánchez-Carnerero E. M. Moreno F. Maroto B. L. Agarrabeitia A. R. Ortiz M. J. Vo B. G.. (2014). Circularly polarized luminescence by visible-light absorption in a chiral O-BODIPY dye: Unprecedented design of CPL organic molecules from achiral chromophores. J. Am. Chem. Soc. 136, 3346–3349. 10.1021/ja412294s 24524257 PMC3984031

[B70] Santoro F. Barone V. (2010). Computational approach to the study of the lineshape of absorption and electronic circular dichroism spectra. Int. J. Quantum Chem. 110, 476–486. 10.1002/qua.22197

[B71] Santoro F. Improta R. Lami A. Bloino J. Barone V. (2007a). Effective method to compute Franck-Condon integrals for optical spectra of large molecules in solution. J. Chem. Phys. 126:084509. 10.1063/1.2437197 17343460

[B72] Santoro F. Lami A. Improta R. Barone V. (2007b). Effective method to compute vibrationally resolved optical spectra of large molecules at finite temperature in gas phase and in solution. J. Chem. Phys. 126:184102. 10.1063/1.2721539 17508787

[B73] Santoro F. Lami A. Improta R. Bloino J. Barone V. (2008). Effective method for the computation of optical spectra of large molecules at finite temperature including the Duschinsky and Herzberg-Teller effect: the q_*X*_ band of porphyrin as a case study. J. Chem. Phys. 128:224311. 10.1063/1.2929846 18554017

[B74] Sharp T. E. Rosenstock H. M. (1963). Franck–Condon factors for polyatomic molecules. J. Chem. Phys. 41, 3453–3463. 10.1063/1.1725748

[B75] Shivran N. Tyagi M. Mula S. Gupta P. Saha B. Patro B. S.. (2016). Syntheses and photodynamic activity of some glucose-conjugated BODIPY dyes. Eur. J. Med. Chem. 122, 352–365. 10.1016/j.ejmech.2016.06.050 27393947

[B76] Swart M. Bickelhaupt F. M. (2006). Optimization of strong and weak coordinates. Int. J. Quantum Chem. 106, 2536–2544. 10.1002/qua.21049

[B77] Telea A. C. (2007). Data Visualization: Principles and Practice. Boca Raton, FL: AK Peters; CRC Press.

[B78] Turksoy A. Yildiz D. Akkaya E. U. (2019). Photosensitization and controlled photosensitization with BODIPY dyes. Coord. Chem. Rev. 379, 47–64. 10.1016/j.ccr.2017.09.029

[B79] Ulrich G. Ziessel R. Harriman A. (2008). The chemistry of fluorescent BODIPY dyes: versatility unsurpassed. Angew. Chem. Int. Ed. 47, 1184–1201. 10.1002/anie.200702070 18092309

[B80] Valero R. Costa R. Moreira I. D. P. R. Truhlar D. G. Illas F. (2008). Performance of the M06 family of exchange-correlation functionals for predicting magnetic coupling in organic and inorganic molecules. J. Chem. Phys. 128:114103. 10.1063/1.2838987 18361550

[B81] Vydrov O. A. Scuseria G. E. (2006). Assessment of a long-range corrected hybrid functional. J. Chem. Phys. 125:234109. 10.1063/1.2409292 17190549

[B82] Wang T. Douglass E. F Jr Fitzgerald K. J. Spiegel D. A. (2013). A “turn-on” fluorescent sensor for methylglyoxal. J. Am. Chem. Soc. 135, 12429–12433. 10.1021/ja406077j 23931147

[B83] Yanai T. Tew D. P. Handy N. C. (2004). A new hybrid exchange–correlation functional using the coulomb-attenuating method (CAM-B3LYP). Chem. Phys. Lett. 393, 51–57. 10.1016/j.cplett.2004.06.01127843488

[B84] Zhang X. Liu Y. Xu C. Teng L. Liu H. Ren T.-B.. (2020). pH stimuli-disaggregated BODIPY: an activated photodynamic/photothermal sensitizer applicable to tumor ablation. Chem. Commun. (Camb). 56, 1956–1959. 10.1039/C9CC09790B 31956868

[B85] Zhao J. Xu K. Yang W. Wang Z. Zhong F. (2015). The triplet excited state of BODIPY: formation, modulation and application. Chem. Soc. Rev. 44, 8904–8939. 10.1039/C5CS00364D 26465741

[B86] Zinna F. Bruhn T. Guido C. A. Ahrens J. Bröring M. Di Bari L.. (2016). Circularly polarized luminescence from axially chiral BODIPY DYEmers: an experimental and computational study. Chem. Eur. J. 22, 16089–16098. 10.1002/chem.201602684 27658919

[B87] Zinna F. Di Bari L. (2015). Lanthanide circularly polarized luminescence: bases and applications. Chirality 27, 1–13. 10.1002/chir.22382 25318867

