# Supplementary material for: Theoretical Investigation of the Circularly Polarized Luminescence of a Chiral Boron Dipyrromethene (BODIPY) Dye
Source: Front Chem. 2020 Sep 15;8:801. doi: 10.3389/fchem.2020.00801 (PMC7522172; doi:10.3389/fchem.2020.00801)
Supplement: Supplementary file 1 [file Data_Sheet_1.PDF]

Supplementary Material

1 SUPPLEMENTARY TABLES AND FIGURES

1.1 Tables

Table S1. Boltzmann populations of PT and PC at ground state, S_1 and $^{S_2}S_1$ excited states. S_2 in superscript refers to the relative position of the electronic states at the equilibrium geometry of the ground state, after crossing, S_2 becomes the lowest and emitting state.

	S_0	S_1	$^{S_2}S_1$
PC	51.58	49.85	48.15
PT	48.42	50.15	51.85

Table S2. Excited states characteristics of PT conformer at S_0 and $^{S_2}S_1$ geometry. S_2 in superscript refers to the relative position of the electronic states at the equilibrium geometry of the ground state, after crossing, S_2 becomes the lowest and emitting state.

		At S_0 geometry	
Excited State	Energy(eV)	Transition (% weight)	oscillator strengths
1	2.6385	HOMO → LUMO (97)	0.7094
2	3.2125	HOMO-1 → LUMO (95)	0.0058
		At $^{S_2}S_1$ geometry	
Excited State	Energy(eV)	Transition (% weight)	oscillator strengths
1	2.4081	HOMO → LUMO (95)<br>HOMO-1 → LUMO (4)	0.5968
2	2.9983	HOMO-1 → LUMO (92)<br>HOMO-1 → LUMO (4)	0.0192

Table S3. Fragment contributions to the dipole transition moments $S_1 \leftarrow S_0$ at the ground state geometry (cgs units). The values were obtained by numerical integration of the ECTD cube files. Results obtained from TD-DFT calculations are labeled as "Total".

$S_1 \leftarrow S_0$							
PT				PC			
Fragment 1: BINOL							
$\boldsymbol{\mu}_1$	-6.33543E-20	5.82525E-19	1.37994E-19	$\boldsymbol{\mu}_1$	7.60923E-20	-5.90879E-19	-1.10007E-19
$\boldsymbol{m}_1$	1.78114E-22	3.65270E-22	1.12092E-21	$\boldsymbol{m}_1$	-1.91982E-22	-4.30068E-22	-1.08333E-21
Fragment 2: BODIPY							
$\boldsymbol{\mu}_2$	-4.61781E-18	-7.41046E-18	-2.06872E-18	$\boldsymbol{\mu}_2$	4.52078E-18	7.61531E-18	1.59185E-18
$\boldsymbol{m}_2$	-5.76578E-21	-3.44555E-21	2.43235E-20	$\boldsymbol{m}_2$	3.60837E-21	2.94834E-21	-2.50366E-20
Total							
$\boldsymbol{\mu}$	-4.68202E-18	-6.82503E-18	-1.92857E-18	$\boldsymbol{\mu}$	4.59693E-18	7.02475E-18	1.48141E-18
$\boldsymbol{m}$	-5.58853E-21	-3.07770E-21	2.54442E-20	$\boldsymbol{m}$	3.41672E-21	2.51816E-21	-2.61198E-20
RS		DS		RS		DS	
$\boldsymbol{\mu}_1\boldsymbol{m}_1$	3.56174E-40	$\boldsymbol{\mu}_1\boldsymbol{\mu}_1$	3.62392E-37	$\boldsymbol{\mu}_1\boldsymbol{m}_1$	3.58684E-40	$\boldsymbol{\mu}_1\boldsymbol{\mu}1$	3.67030E-37
$\boldsymbol{\mu}_1\boldsymbol{m}_2$	1.71466E-39	$\boldsymbol{\mu}_1\boldsymbol{\mu}_2$	-4.30969E-36	$\boldsymbol{\mu}_1\boldsymbol{m}_2$	1.28666E-39	$\boldsymbol{\mu}_1\boldsymbol{\mu}2$	-4.33084E-36
$\boldsymbol{\mu}_2\boldsymbol{m}_1$	-5.84818E-39	$\boldsymbol{\mu}_2\boldsymbol{\mu}_1$	-4.30969E-36	$\boldsymbol{\mu}_2\boldsymbol{m}_1$	-5.86751E-39	$\boldsymbol{\mu}_2\boldsymbol{\mu}1$	-4.33084E-36
$\boldsymbol{\mu}_2\boldsymbol{m}_2$	1.83977E-39	$\boldsymbol{\mu}_2\boldsymbol{\mu}_2$	8.05187E-35	$\boldsymbol{\mu}_2\boldsymbol{m}_2$	-1.08941E-39	$\boldsymbol{\mu}_2\boldsymbol{\mu}2$	8.09643E-35
sum.	-1.93757E-39	sum.	7.22617E-35	sum.	-5.31157E-39	sum.	7.26697E-35
tot.	-1.89995E-39	tot.	7.22218E-35	tot.	-5.29833E-39	tot.	7.26735E-35

Table S4. Fragment contributions to the dipole transition moments $S_1 \rightarrow S_0$ at the S_1 state geometry before crossing (cgs units). The values were obtained by numerical integration of the ECTD cube files. Results obtained from TD-DFT calculations are labeled as "Total".

$S_1 \rightarrow S_0$ before crossing							
PT				PC			
Fragment 1: BINOL							
$\boldsymbol{\mu}_1$	6.94782E-20	-5.48119E-19	-1.41823E-19	$\boldsymbol{\mu}_1$	-7.18971E-20	5.51815E-19	1.16850E-19
$\boldsymbol{m}_1$	-1.38608E-22	-2.77099E-22	-1.04601E-21	$\boldsymbol{m}_1$	1.56391E-22	3.41948E-22	9.98971E-22
Fragment 2: BODIPY							
$\boldsymbol{\mu}_2$	4.81377E-18	7.04679E-18	2.04589E-18	$\boldsymbol{\mu}_2$	-4.77945E-18	-7.24218E-18	-1.58661E-18
$\boldsymbol{m}_2$	6.28599E-21	2.25116E-21	-2.16215E-20	$\boldsymbol{m}_2$	-4.25745E-21	-2.01242E-21	2.24710E-20
Total							
$\boldsymbol{\mu}$	-6.01594E-19	-7.97208E-18	-2.40568E-18	$\boldsymbol{\mu}$	-4.85079E-18	-6.69138E-18	-1.47029E-18
$\boldsymbol{m}$	1.50733E-21	-6.18690E-21	2.02689E-20	$\boldsymbol{m}$	-4.10123E-21	-1.67207E-21	2.34718E-20
RS		DS		RS		DS	
$\boldsymbol{\mu}_1\boldsymbol{m}_1$	2.90601E-40	$\boldsymbol{\mu}_1\boldsymbol{\mu}_1$	3.25375E-37	$\boldsymbol{\mu}_1\boldsymbol{m}_1$	2.94178E-40	$\boldsymbol{\mu}_1\boldsymbol{\mu}1$	3.23323E-37
$\boldsymbol{\mu}_1\boldsymbol{m}_2$	2.26926E-39	$\boldsymbol{\mu}_1\boldsymbol{\mu}_2$	-3.81818E-36	$\boldsymbol{\mu}_1\boldsymbol{m}_2$	1.82136E-39	$\boldsymbol{\mu}_1\boldsymbol{\mu}2$	-3.83811E-36
$\boldsymbol{\mu}_2\boldsymbol{m}_1$	-4.75991E-39	$\boldsymbol{\mu}_2\boldsymbol{\mu}_1$	-3.81818E-36	$\boldsymbol{\mu}_2\boldsymbol{m}_1$	-4.80890E-39	$\boldsymbol{\mu}_2\boldsymbol{\mu}1$	-3.83811E-36
$\boldsymbol{\mu}_2\boldsymbol{m}_2$	1.88774E-39	$\boldsymbol{\mu}_2\boldsymbol{\mu}_2$	7.70153E-35	$\boldsymbol{\mu}_2\boldsymbol{m}_2$	-7.30297E-40	$\boldsymbol{\mu}_2\boldsymbol{\mu}2$	7.78097E-35
sum.	-3.12309E-40	sum.	6.97044E-35	sum.	-3.42366E-39	sum.	7.04568E-35
tot.	-3.44896E-40	tot.	6.97033E-35	tot.	-3.42749E-39	tot.	7.04665E-35

Table S5. Fragment contributions to the dipole transition moments $S_1 \rightarrow S_0$ at the lowest excited state geometry after crossing (cgs units). The values were obtained by numerical integration of the ECTD cube files. Results obtained from TD-DFT calculations are labeled as “Total”.

$S_1 \rightarrow S_0$ after crossing							
PT				PC			
Fragment 1: BINOL							
μ_1	-4.51402E-19	-3.55003E-19	-2.45944E-19	μ_1	6.56755E-20	-5.89861E-19	-1.79409E-19
m_1	7.97052E-24	-7.79176E-23	-1.15090E-21	m_1	-1.22524E-22	-2.28036E-22	-9.70905E-22
Fragment 2: BODIPY							
μ_2	6.77521E-19	8.18222E-18	2.57691E-18	μ_2	5.35912E-18	6.54588E-18	1.73424E-18
m_2	-2.06461E-21	6.47339E-21	-1.87927E-20	m_2	4.96888E-21	1.70772E-21	-2.16946E-20
Total							
μ	2.26515E-19	7.82682E-18	2.33147E-18	μ	5.42412E-18	5.95672E-18	1.55303E-18
m	-2.05684E-21	6.39558E-21	-1.99433E-20	m	4.84466E-21	1.47739E-21	-2.26643E-20
RS		DS		RS		DS	
$\mu_1 m_1$	3.07120E-40	$\mu_1 \mu_1$	3.90279E-37	$\mu_1 m_1$	3.00652E-40	$\mu_1 \mu 1$	3.84437E-37
$\mu_1 m_2$	3.25585E-39	$\mu_1 \mu_2$	-3.84432E-36	$\mu_1 m_2$	3.21123E-39	$\mu_1 \mu 2$	-3.82033E-36
$\mu_2 m_1$	-3.59791E-39	$\mu_2 \mu_1$	-3.84432E-36	$\mu_2 m_1$	-3.83310E-39	$\mu_2 \mu 1$	-3.82033E-36
$\mu_2 m_2$	3.14084E-39	$\mu_2 \mu_2$	7.40483E-35	$\mu_2 m_2$	1.83710E-40	$\mu_2 \mu 2$	7.45762E-35
sum.	3.10590E-39	sum.	6.67500E-35	sum.	-1.37502E-40	sum.	6.73200E-35
tot.	3.09381E-39	tot.	6.67461E-35	tot.	-1.20058E-40	tot.	6.73155E-35

1.2 Figures

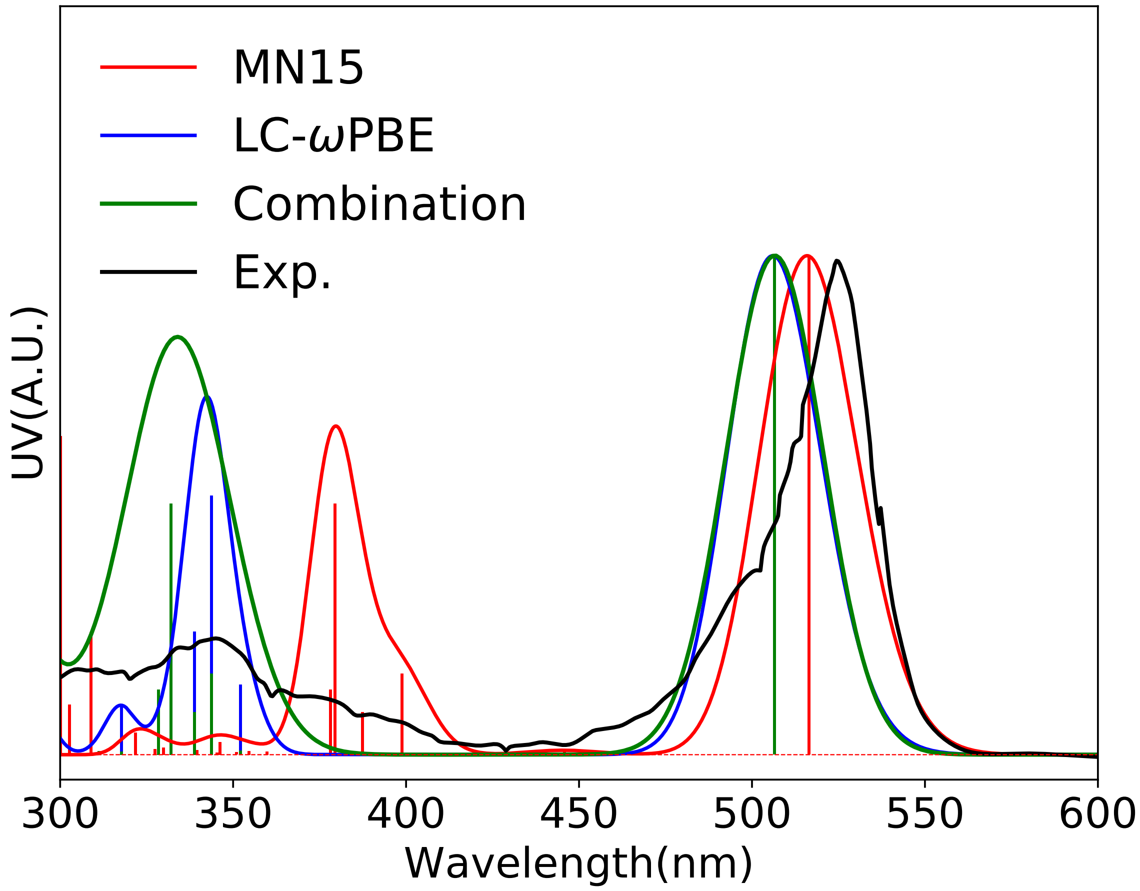

Figure S1. Pure electronic $S_1 \leftarrow S_0$ OPA spectrum of (*R*)-O-BODIPY at the LC-ωPBE and MN15 levels in conjunction with the 6-31+G(d) basis set. The "Combination" spectrum is obtained by computing the MN15/6-31+G(d) spectrum with the electronic transition energies obtained at the LC-ωPBE/6-31+G(d) level. The stick spectra correspond to the electronic transitions, arbitrarily scaled to fit the picture.

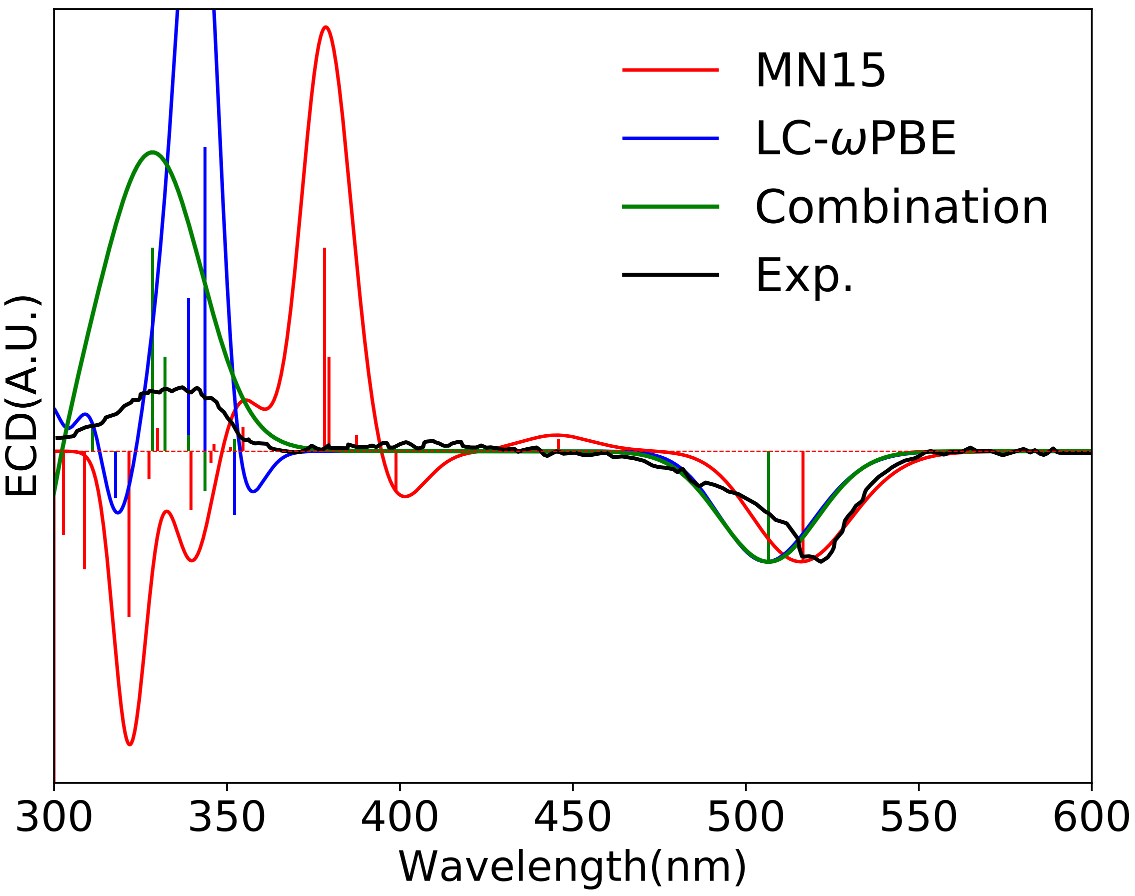

Figure S2. Pure electronic $S_1 \leftarrow S_0$ ECD spectrum of (*R*)-O-BODIPY at the LC-ωPBE and MN15 levels in conjunction with the 6-31+G(d) basis set. The "Combination" spectrum is obtained by computing the MN15/6-31+G(d) spectrum with the electronic transition energies obtained at the LC-ωPBE/6-31+G(d) level. The stick spectra correspond to the electronic transitions, arbitrarily scaled to fit the picture.

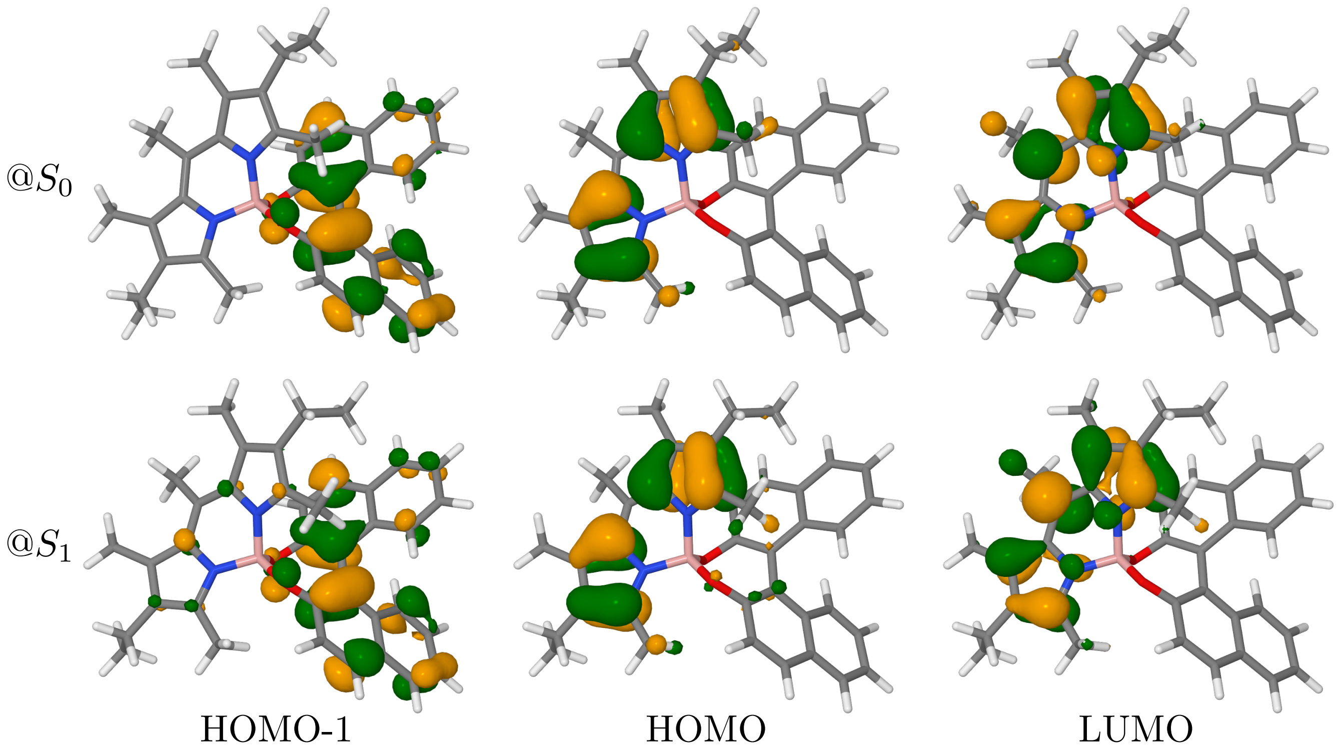

Figure S3. Frontiers molecular orbitals of PC conformer at S_0 and S_1 geometries (isodensity surfaces at $\pm$ 0.04 $(e/{a_0}^3)^{1/2}$).

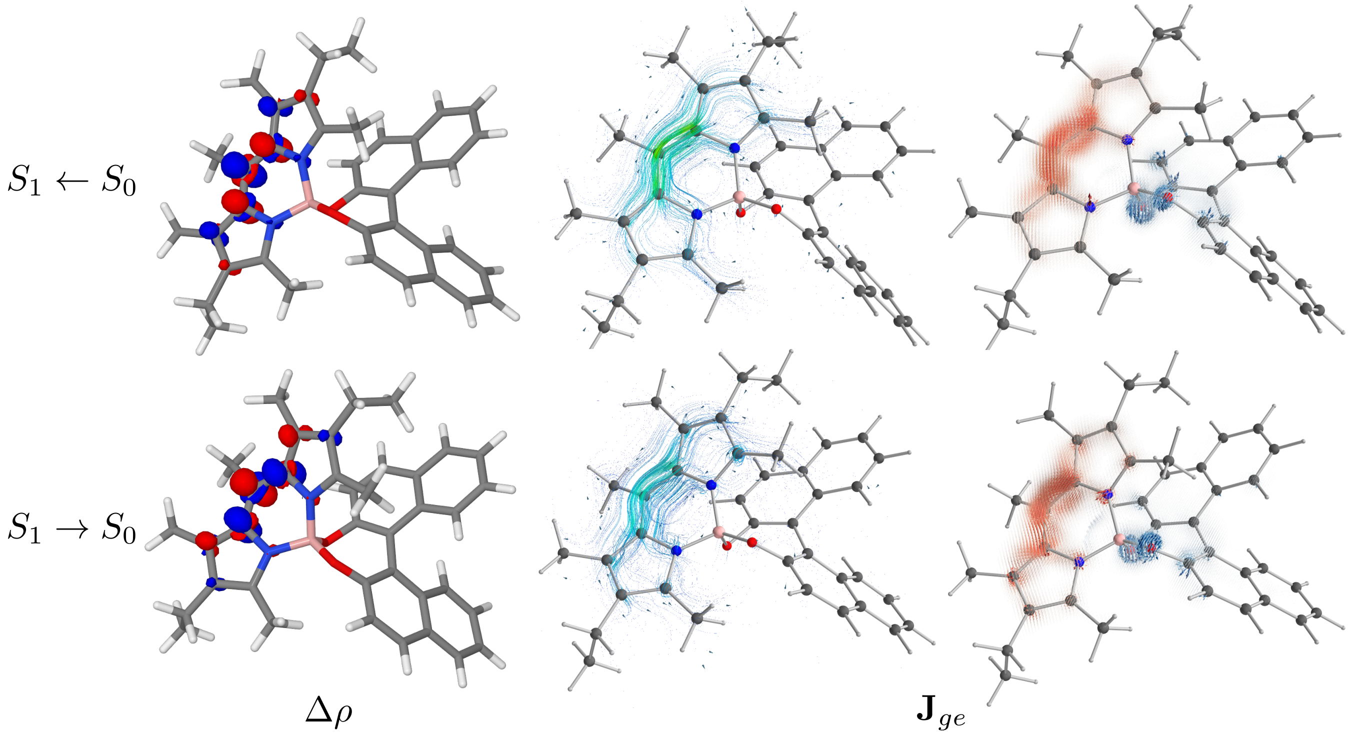

Figure S4. In the left panels the difference between the electronic density of the final and the initial states ($\Delta\rho$) of the PC conformer is represented as isosurfaces (isodensity surfaces at $\pm$ 0.004 $(e/{a_0}^3)$). In the central panels the ETCD ($\mathbf{J}_{ge}$ vector fields are represented by means of streamline objects. In the right panels, the ETCDs have been partitioned between BODIPY and BINOL contributions, with the BINOL field being magnified 15 times. The resulting fields are represented by the means of the Hedgehog representation.

Pre

Post

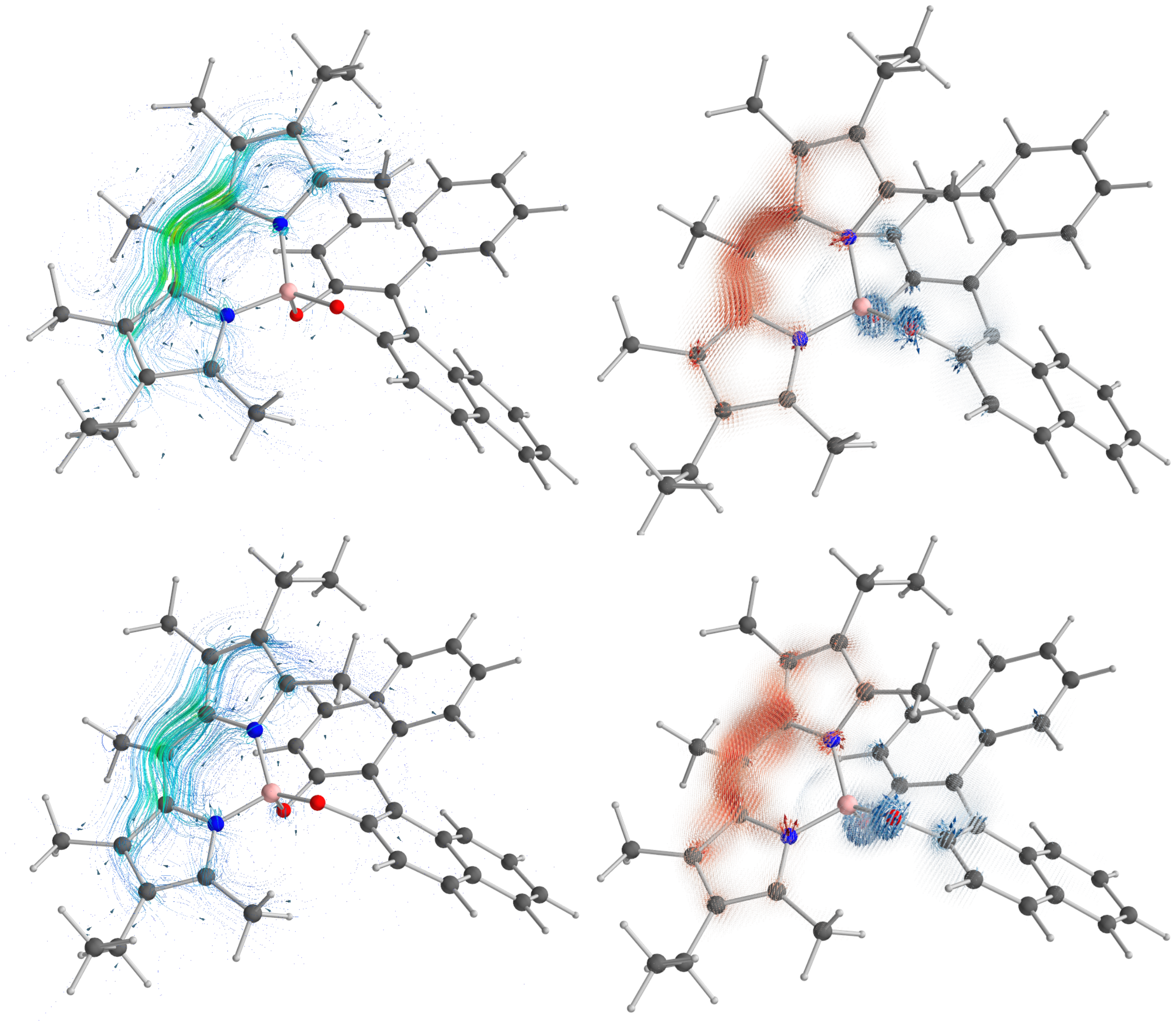

Figure S5. In the top panels are represented the ETCD vector fields of PT the conformer for the transition $S_1 \to S_0$ at the S_1 state geometry before crossing ("Pre"), compared to those for the transition $S_1 \to S_0$ at the lowest excited-state geometry after crossing ("Post"). In the left panels, the ETCD ($\mathbf{J}_{ge}$ vector fields are represented by means of streamline objects. In the right panels, the ETCDs have been partitioned between BODIPY and BINOL contributions, with the BINOL field being magnified 15 times. The resulting fields are represented by the means of the Hedgehog representation.

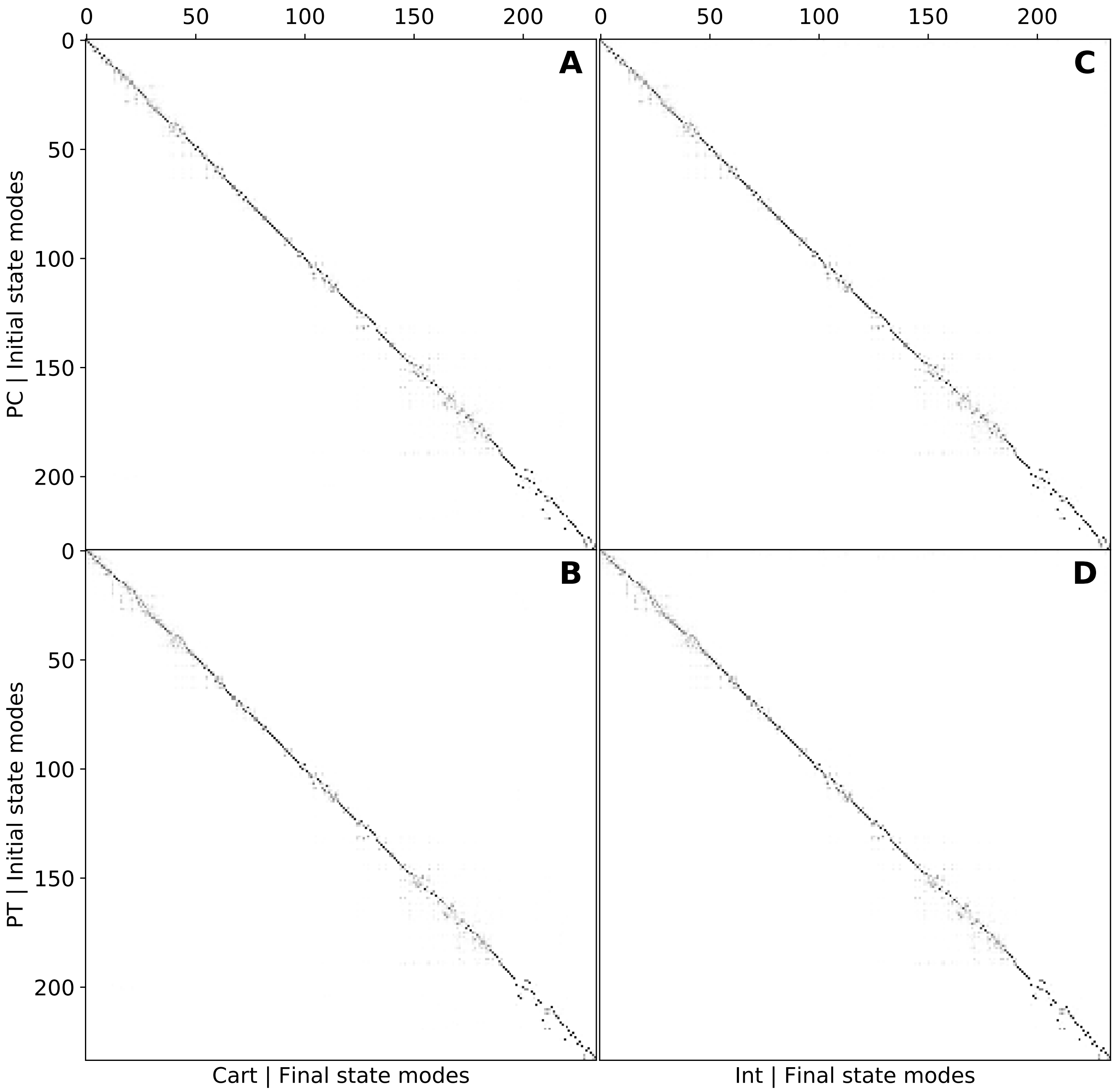

Figure S6. Comparison of the Duschinsky matrices **J** of PC (panels **A** and **C**) and PT (panels **B** and **D**) conformers of (*R*)-O-BODIPY in Cartesian (panels **A** and **B**) and internal (panels **C** and **D**) coordinates for the $S_1 \leftarrow S_0$ OPA spectrum at AH|FC level. A grayscale, from white for $\mathsf{J}_{ik}{}^2 = 0$ to black for $\mathsf{J}_{ik}{}^2 = 1$, was used. To display the mode mixing better, each row was normalized, so the elements are displayed as $\mathsf{J}_{ik}{}^2 / \sum_{l=1}^{N} \mathsf{J}_{il}{}^2$.

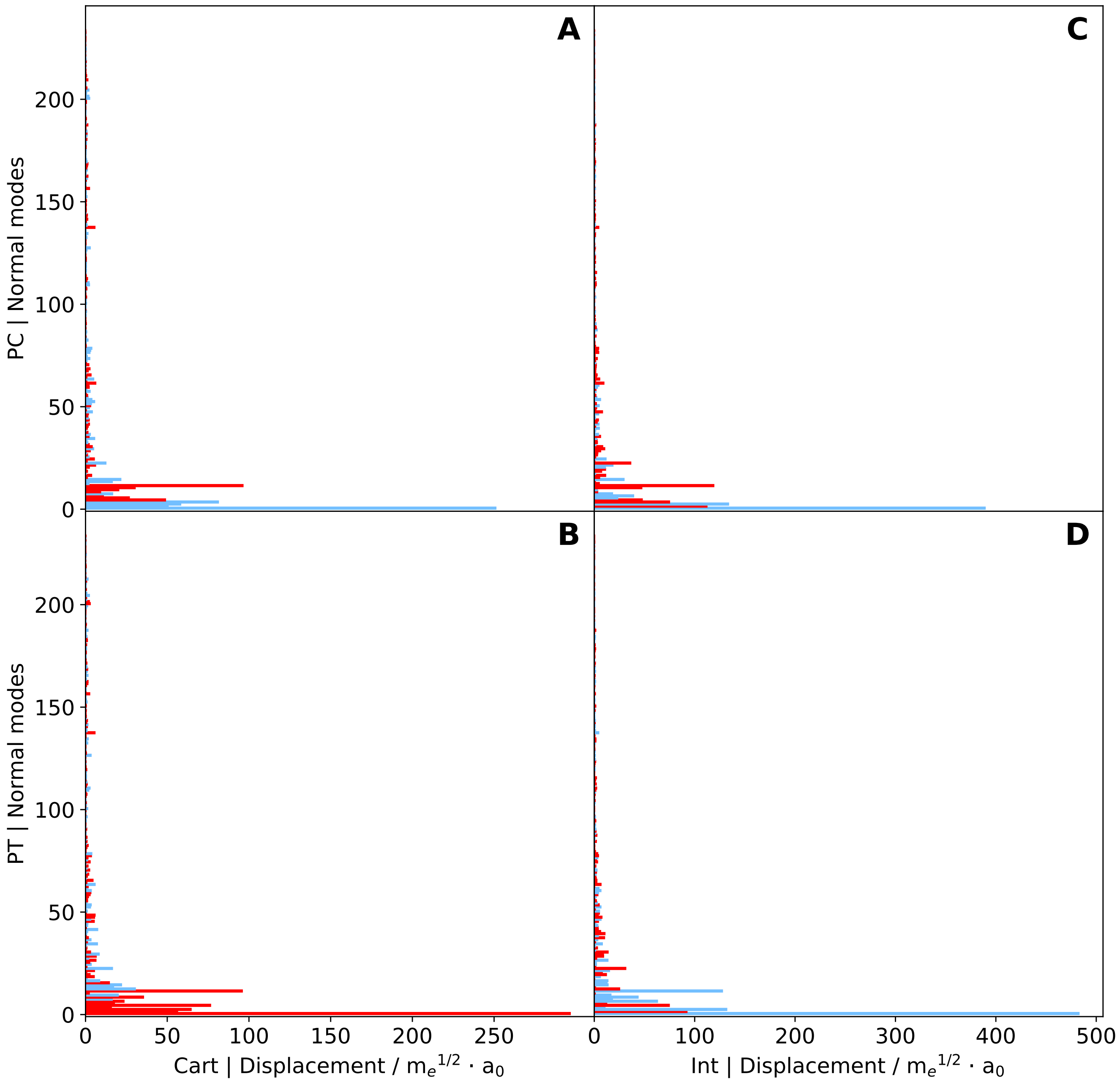

Figure S7. Comparison of the shift vector **K** of PC (panels **A** and **C**) and PT (panels **B** and **D**) conformers of (*R*)-O-BODIPY in Cartesian (panels **A** and **B**) and internal (panels **C** and **D**) coordinates, in atomic units, for the $S_1 \leftarrow S_0$ OPA spectrum at AH|FC level. A blue bar indicates a positive vector, a red one for negative vector.

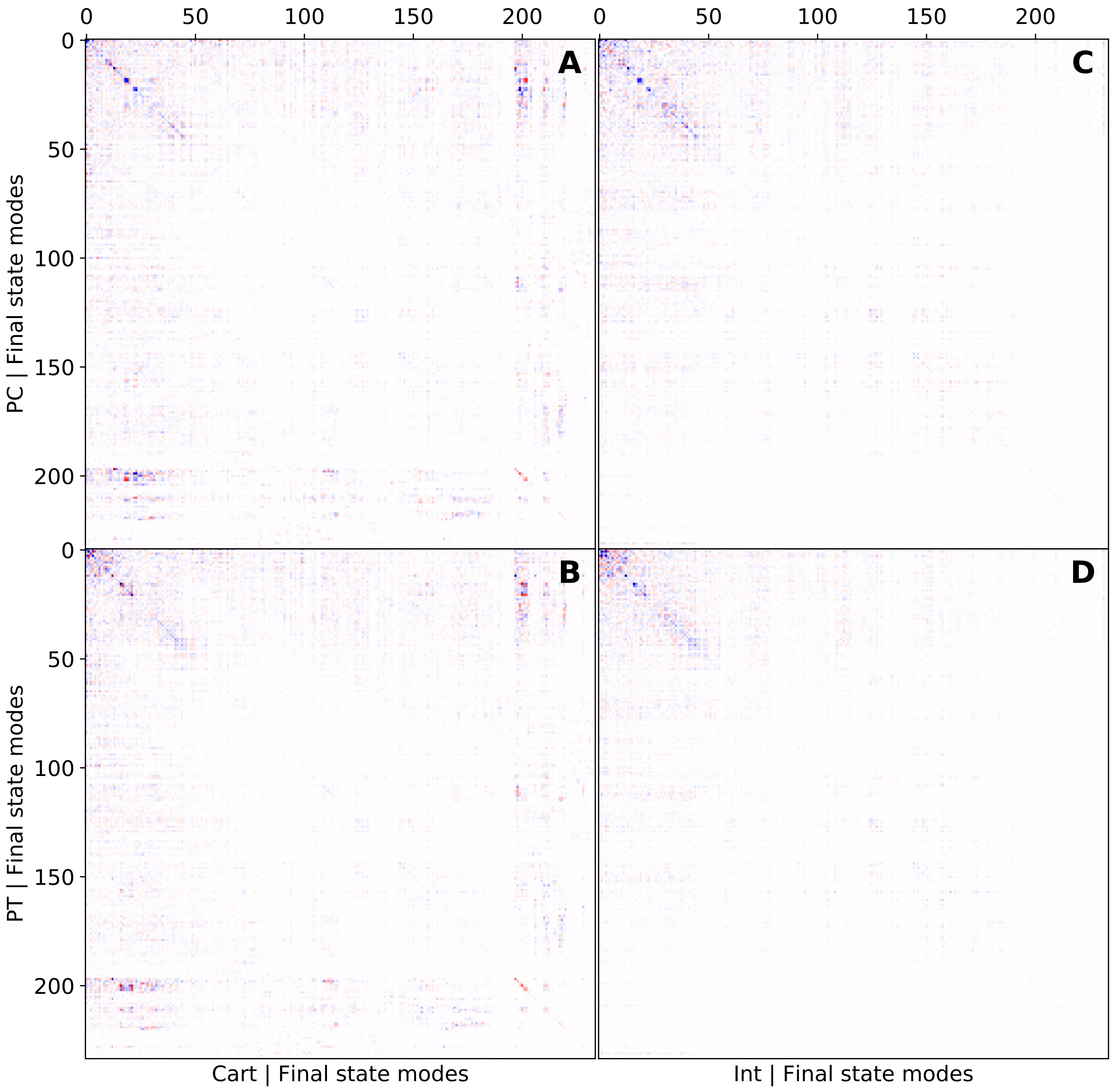

Figure S8. Comparison of the Sharp and Rosenstock **C** matrix(Sharp and Rosenstock, 1963) of PC (panels **A** and **C**) and PT (panels **B** and **D**) conformers of (*R*)-O-BODIPY in Cartesian (panels **A** and **B**) and internal (panels **C** and **D**) coordinates for the $S_1 \leftarrow S_0$ OPA spectrum at AH|FC level. As described in Ref. (Bloino et al., 2016), the C matrix provides qualitative information on the incidence of combination bands over the overtones (vibrational progression). A diagonal matrix means that mainly overtones contribute, while diffuse matrix (non-negligible off-diagonal elements) indicates that combination bands contribute a lot, leading to a more broadened band. Blue squares represent positive elements of C_{ij}, red negative ones. The matrices were normalized so that the highest element in absolute value is set to 1. Normalization factors: **A**: 0.216282, **B**: 0.254269, **C**: 0.158801, **D**: 0.206263

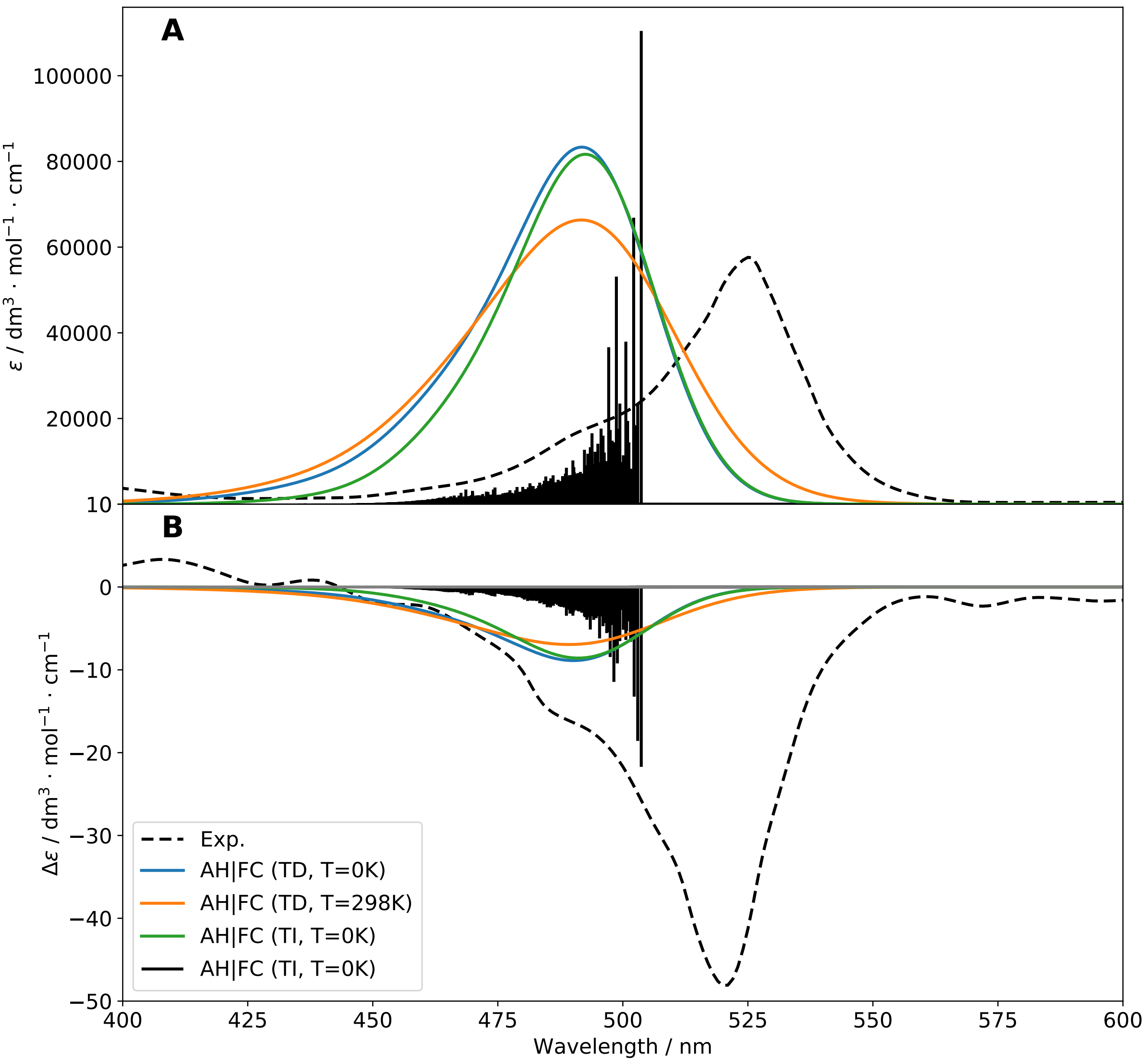

Figure S9. Comparison of the $S_1 \leftarrow S_0$ OPA (panel **A**) and ECD (panel **B**) of (*R*)-O-BODIPY computed at the AH|FCHT level within the sum-over-states (TI, green solid line) and path integral (TD, blue solid line), and with temperature effects (TD, T=298K, orange solid line). Gaussian distribution functions with half-widths at half-maximum of 500 cm^{-1} were used to simulate the experimental broadening. The stick spectrum was computed at T=0K with the sum-over-states approach (black solid line) and arbitrary scaled to fit the figure.

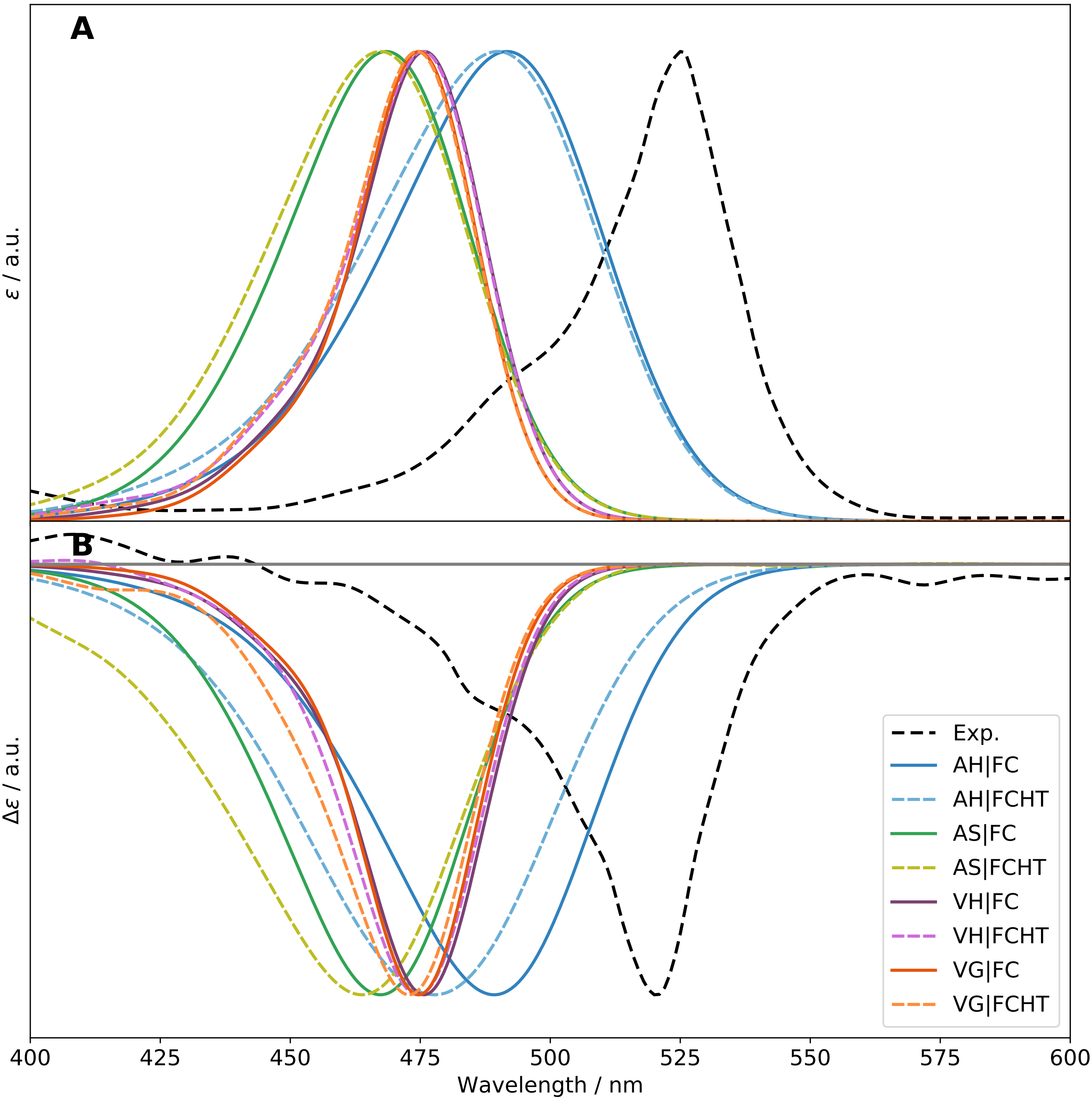

Figure S10. Normalized $S_1 \leftarrow S_0$ OPA (panel **A**) and ECD (panel **B**) spectra of (*R*)-O-BODIPY with different models of vibronic spectra levels within the TD framework at T=298K. Gaussian distribution functions with half-widths at half-maximum of 500 cm^{-1} were used to simulate the experimental broadening.

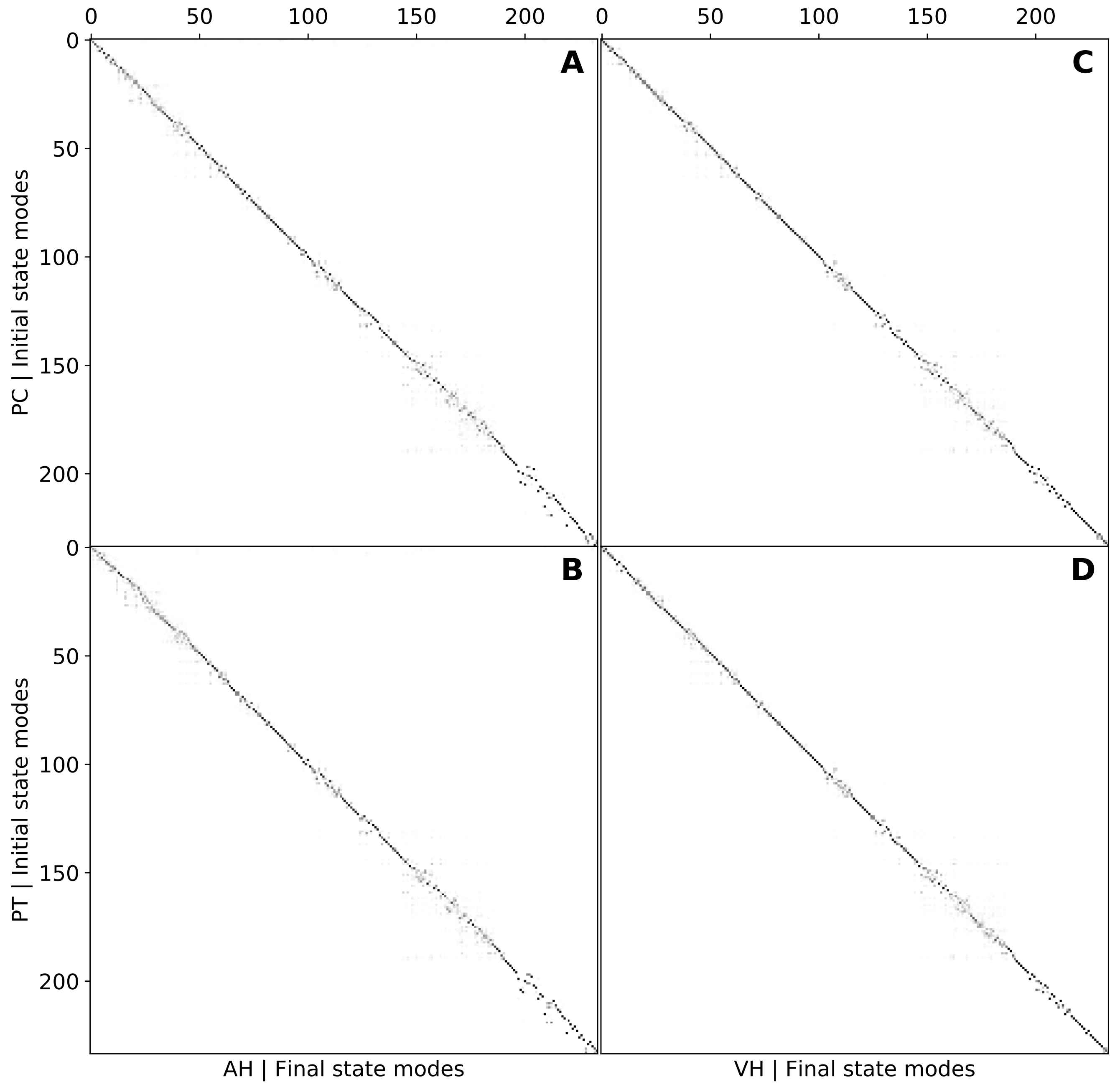

Figure S11. Comparison of the Duschinsky matrices **J** of PC (panels **A** and **C**) and PT (panels **B** and **D**) conformers of (*R*)-O-BODIPY with the AH|FC (panels **A** and **B**) and VH|FC (panels **C** and **D**) models in absorption for the $S_1 \leftarrow S_0$ transition (OPA/ECD). See Fig. S6 for details.

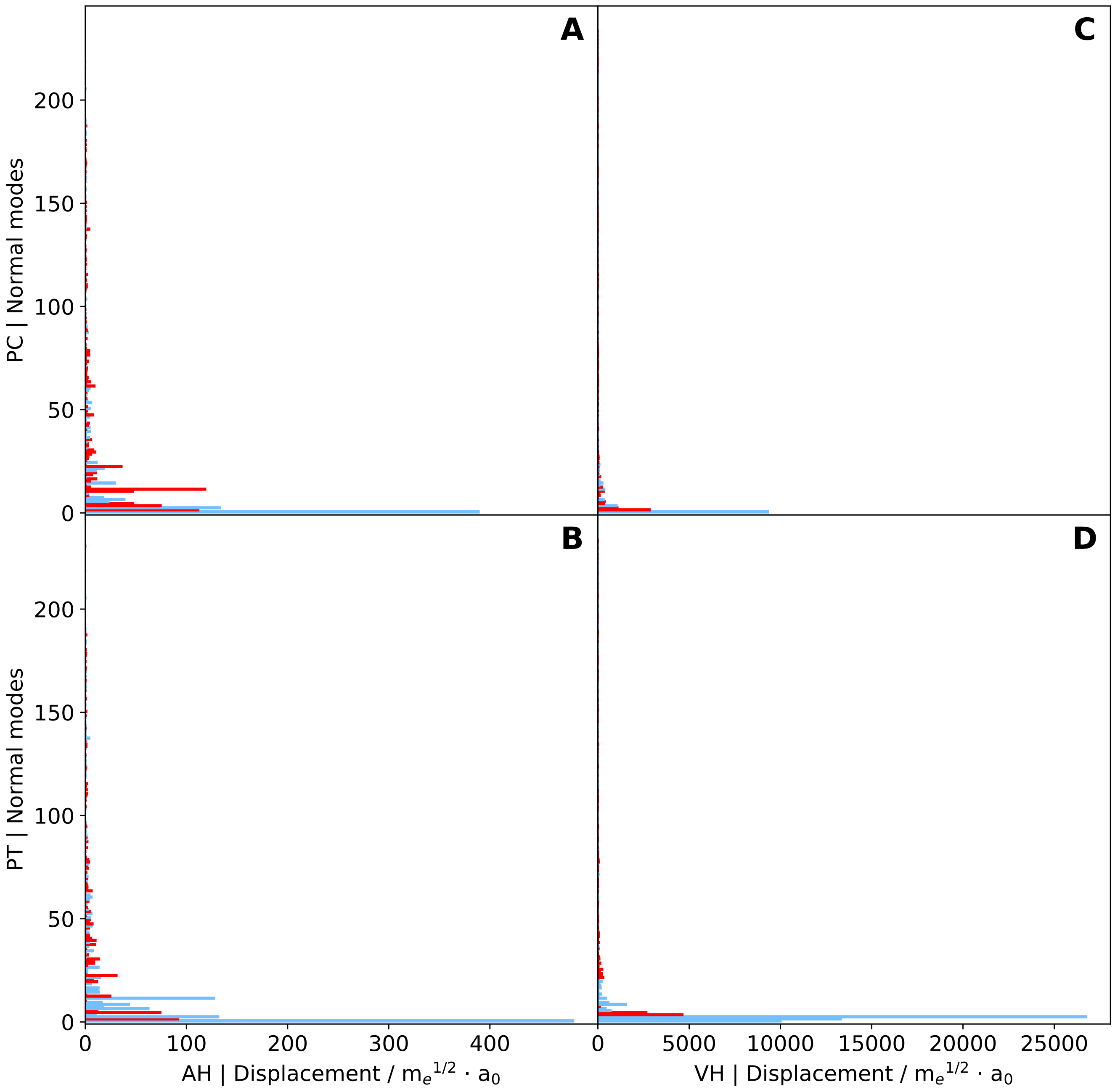

Figure S12. Comparison of the shift vectors **K** of PC (panels **A** and **C**) and PT (panels **B** and **D**) conformers of (*R*)-O-BODIPY with the AH|FC (panels **A** and **B**) and VH|FC (panels **C** and **D**) models in absorption for the $S_1 \leftarrow S_0$ transition (OPA/ECD). See Fig. S7 for details.

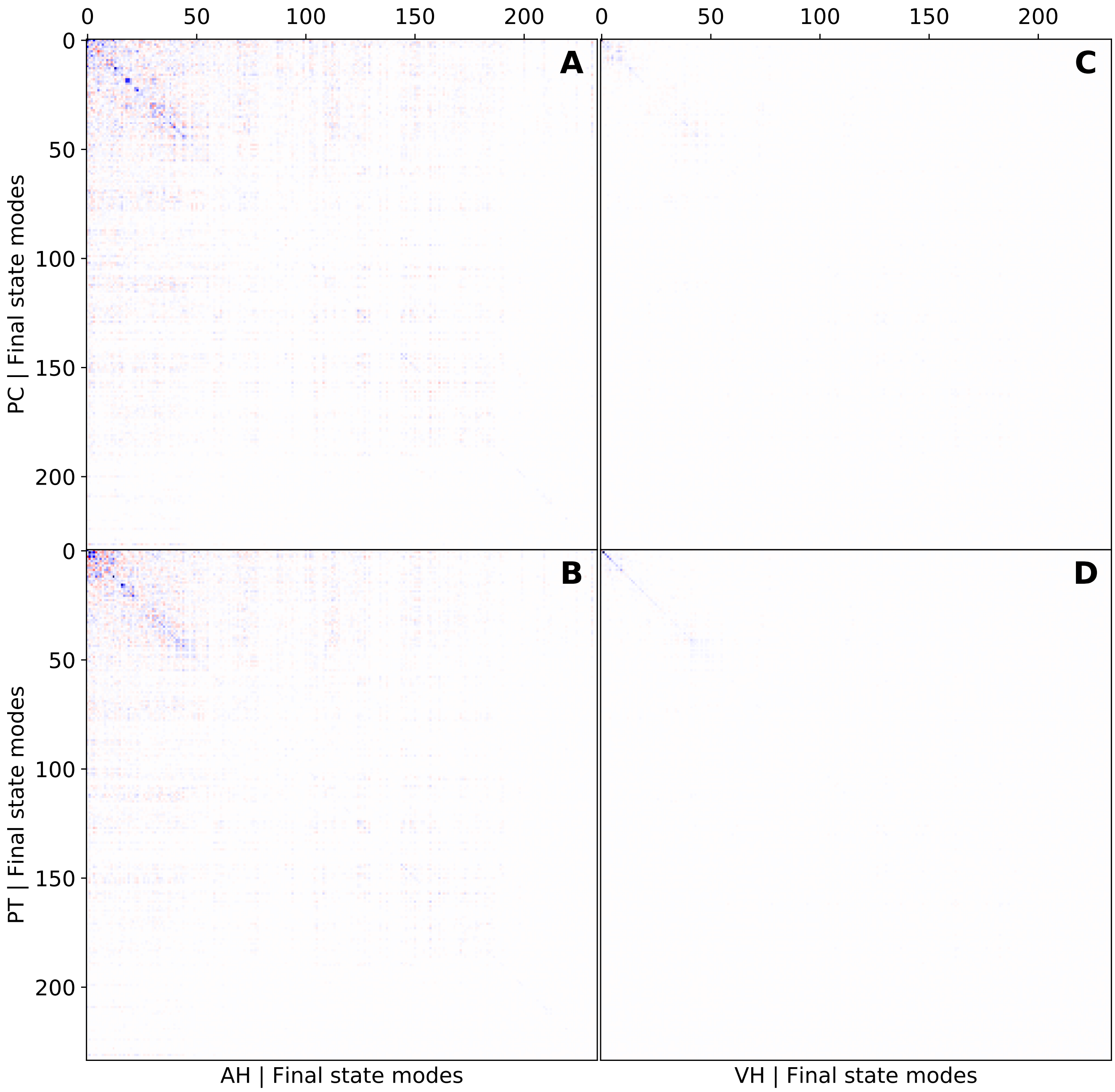

Figure S13. Comparison of the Sharp and Rosenstock **C** matrix(Sharp and Rosenstock, 1963) of PC (panels **A** and **C**) and PT (panels **B** and **D**) conformers of (*R*)-O-BODIPY with the AH|FC (panels **A** and **B**) and VH|FC (panels **C** and **D**) models in absorption for the $S_1 \leftarrow S_0$ transition (OPA/ECD). See Fig. S8 for details. Normalization factors: **A**: 0.158801, **B**: 0.206263, **C**: 0.761068, **D**: 0.910738

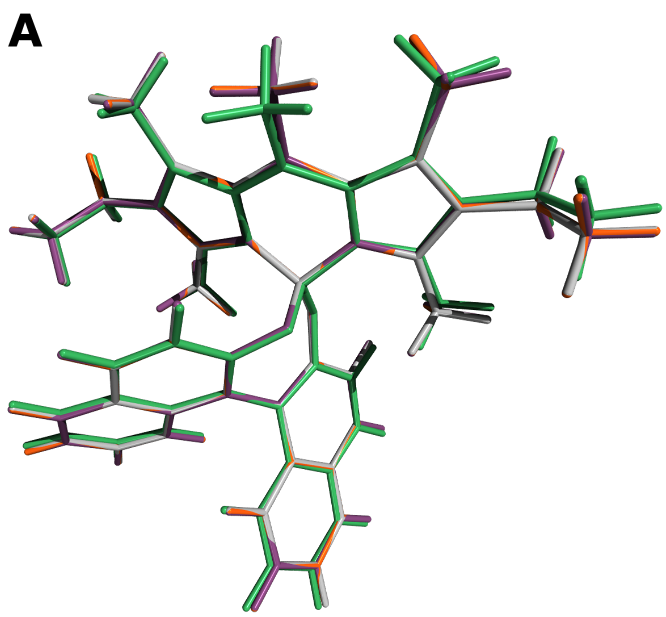

Figure S14. Overlap of the optimized S0 (gray) and S1 (AH/AS, green) geometries, and S1 extrapolated geometries from the VH (purple) and VG (orange) of the PC (panel **A**) and PT (panel **B**) conformers of (*R*)-O-BODIPY

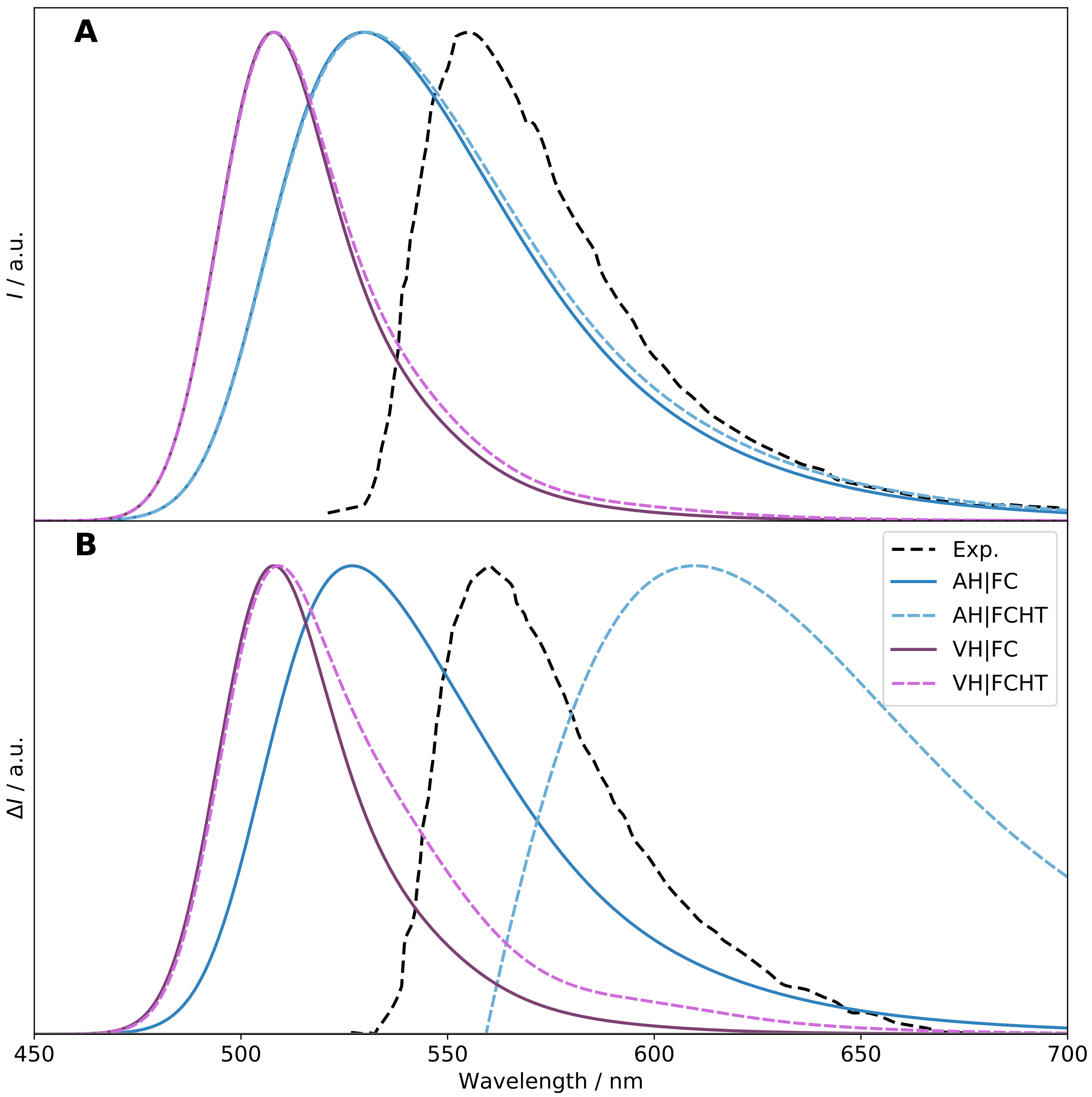

Figure S15. Normalized $S_1 \rightarrow S_0$ OPE (panel **A**) and CPL (panel **B**) spectra of (*R*)-O-BODIPY at the AH|FC, AH|FCHT, VH|FC, VH|FCHT levels within the TD framework at T=298K. Gaussian distribution functions with half-widths at half-maximum of 500 cm^{-1} were used to simulate the experimental broadening.

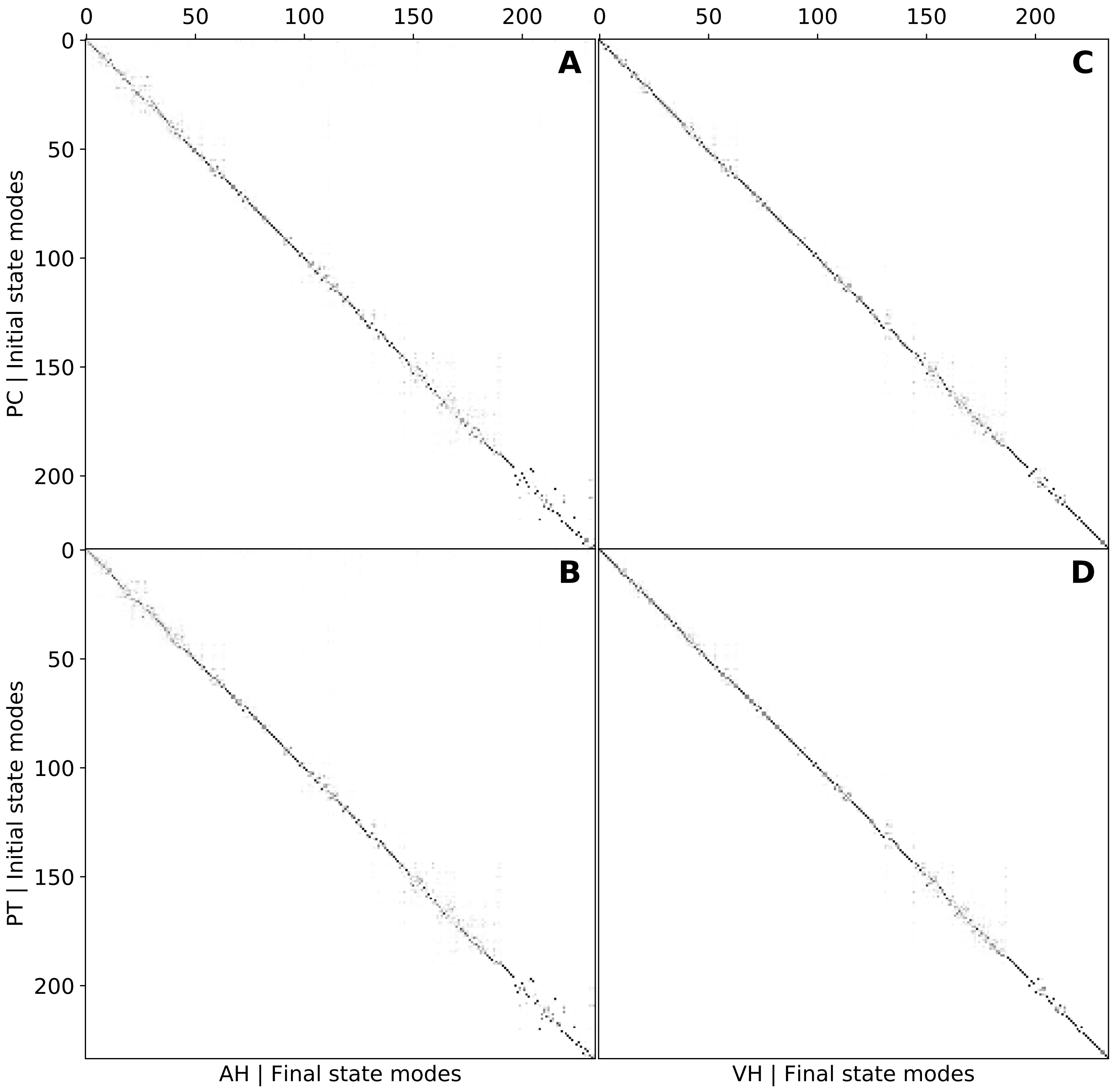

Figure S16. Comparison of the Duschinsky matrices **J** of PC (panels **A** and **C**) and PT (panels **B** and **D**) conformers of (*R*)-O-BODIPY with the AH|FC (panels **A** and **B**) and VH|FC (panels **C** and **D**) models in emission for the $S_1 \rightarrow S_0$ transition (OPE/CPL). See Fig. S6 for details.

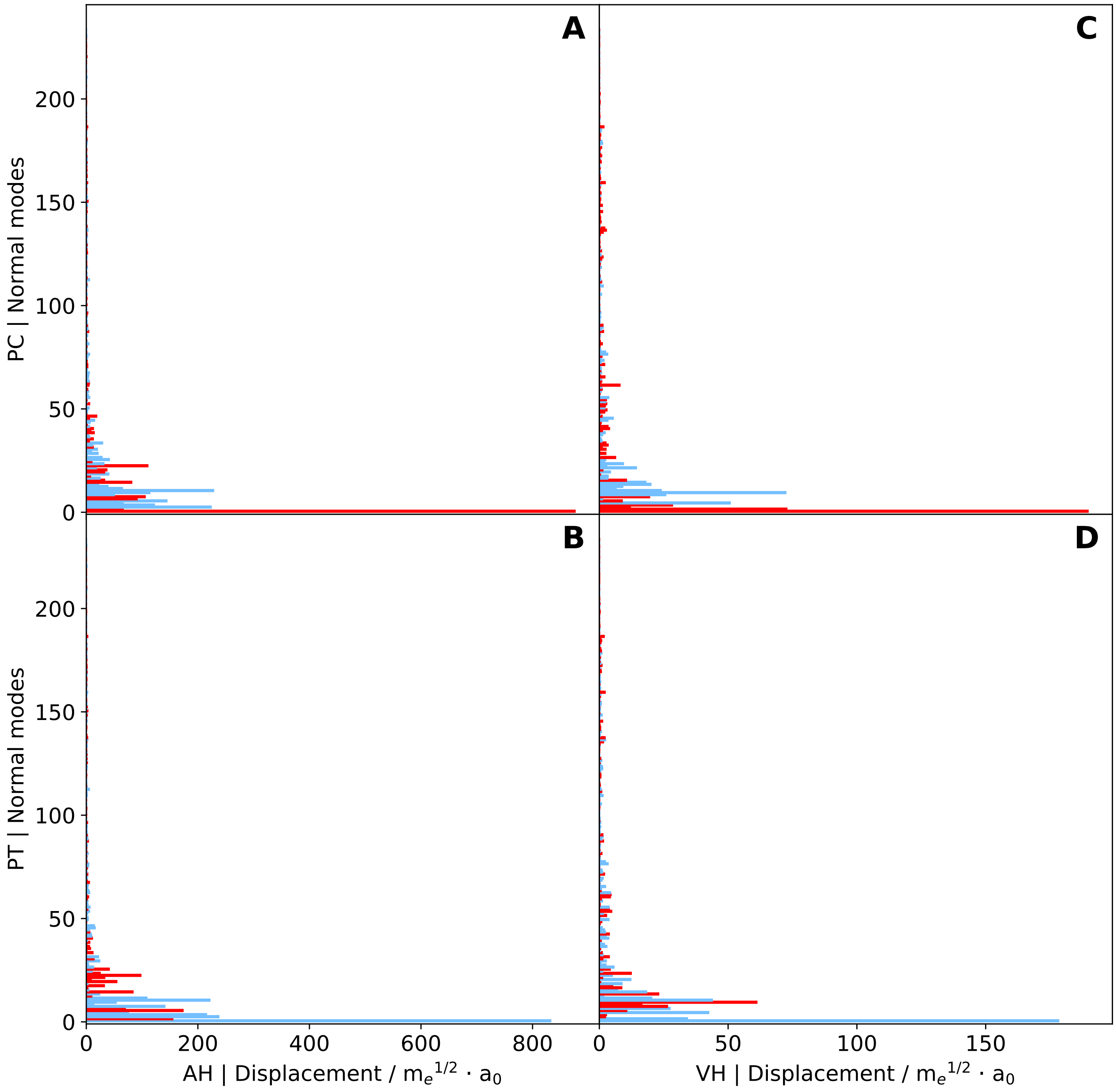

Figure S17. Comparison of the shift vectors **K** of PC (panels **A** and **C**) and PT (panels **B** and **D**) conformers of (*R*)-O-BODIPY with the AH|FC (panels **A** and **B**) and VH|FC (panels **C** and **D**) models in emission for the $S_1 \rightarrow S_0$ transition (OPE/CPL). See Fig. S7 for details.

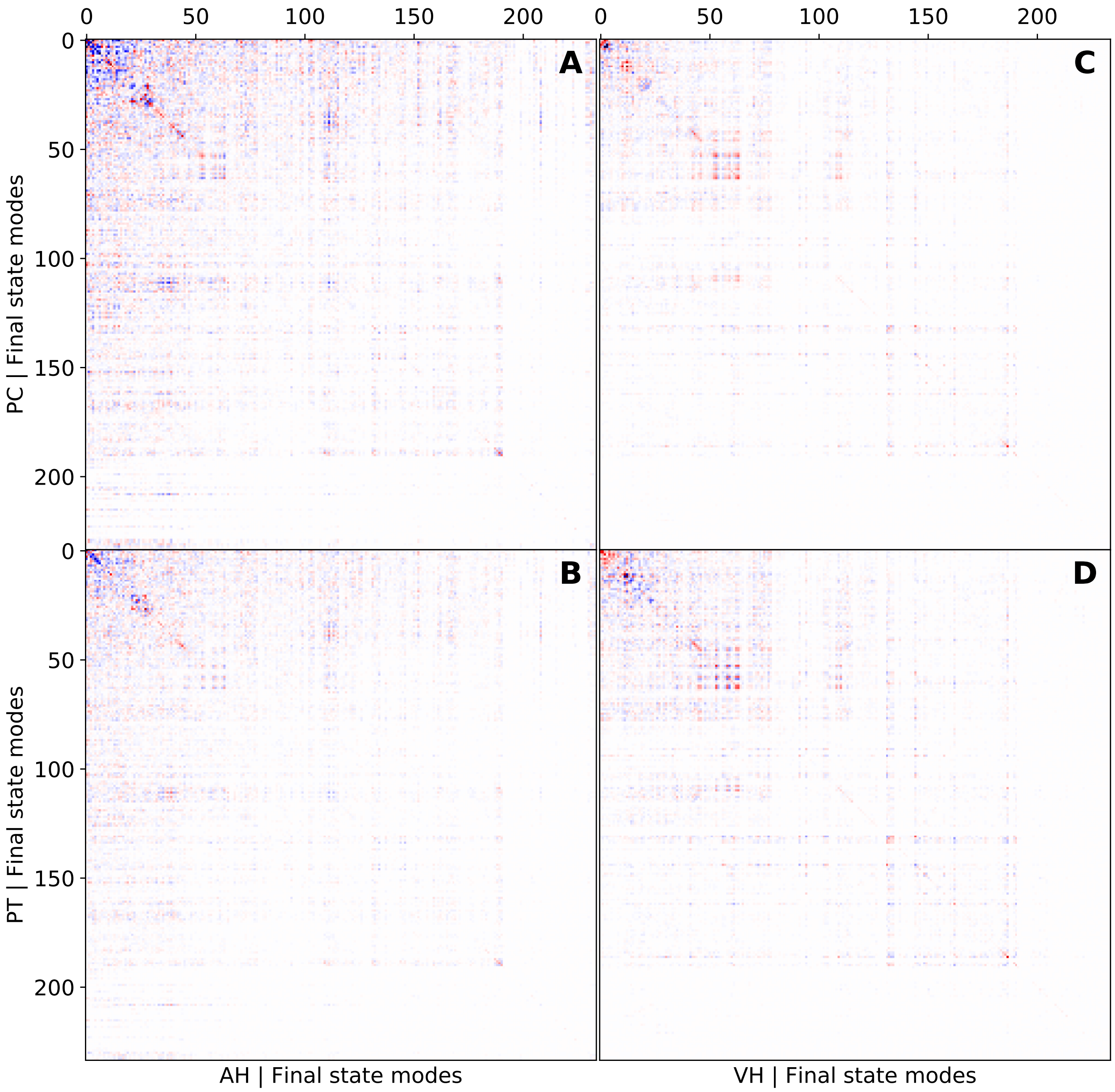

Figure S18. Comparison of the Sharp and Rosenstock **C** matrix(Sharp and Rosenstock, 1963) of PC (panels **A** and **C**) and PT (panels **B** and **D**) conformers of (*R*)-O-BODIPY with the AH|FC (panels **A** and **B**) and VH|FC (panels **C** and **D**) models inemission for the $S_1 \rightarrow S_0$ transition (OPE/CPL). See Fig. S8 for details. Normalization factors: **A**: 0.0980758, **B**: 0.162862, **C**: 0.093715, **D**: 0.0698401

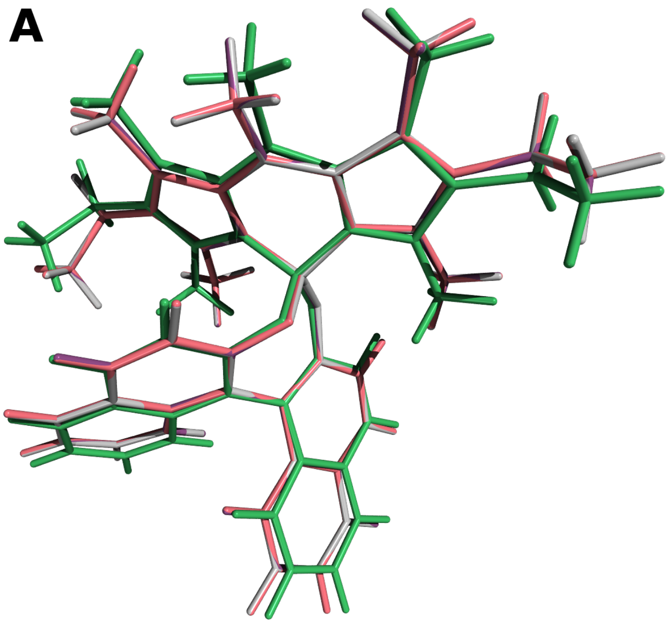

Figure S19. Overlap of the optimized ${}^{S_2}S_1$ (gray) and S_0 (AH/AS, green) geometries, and ${}^{S_2}S_1$ extrapolated geometries from the VH (purple) and VG$_{emi}$ (salmon) of the PC (panel **A**) and PT (panel **B**) conformers of (*R*)-O-BODIPY

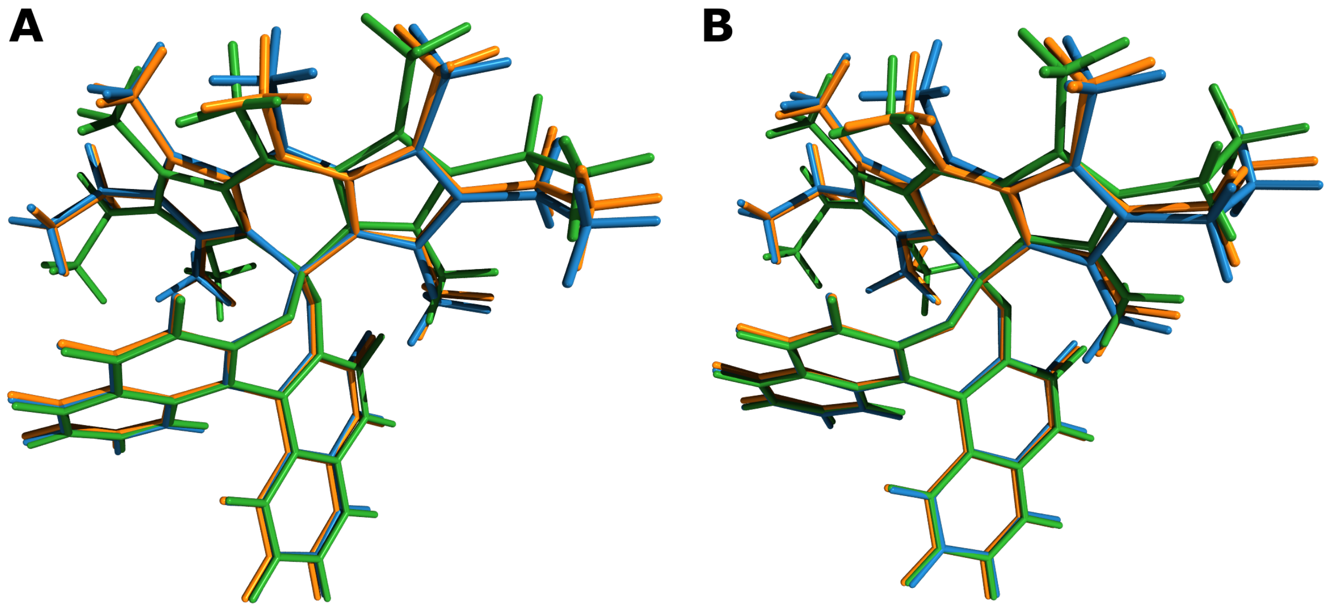

Figure S20. Overlap of the minimum geometries of S_0 (blue), S_1 (orange) and ${}^{S_2}S_1$ (green) of the PC (panel **A**) and PT (panel **B**) conformers of (*R*)-O-BODIPY. S_2 in superscript refers to relative position of the electronic states at the equilibrium geometry of the ground state, after crossing, S_2 becomes the lowest and emitting state.

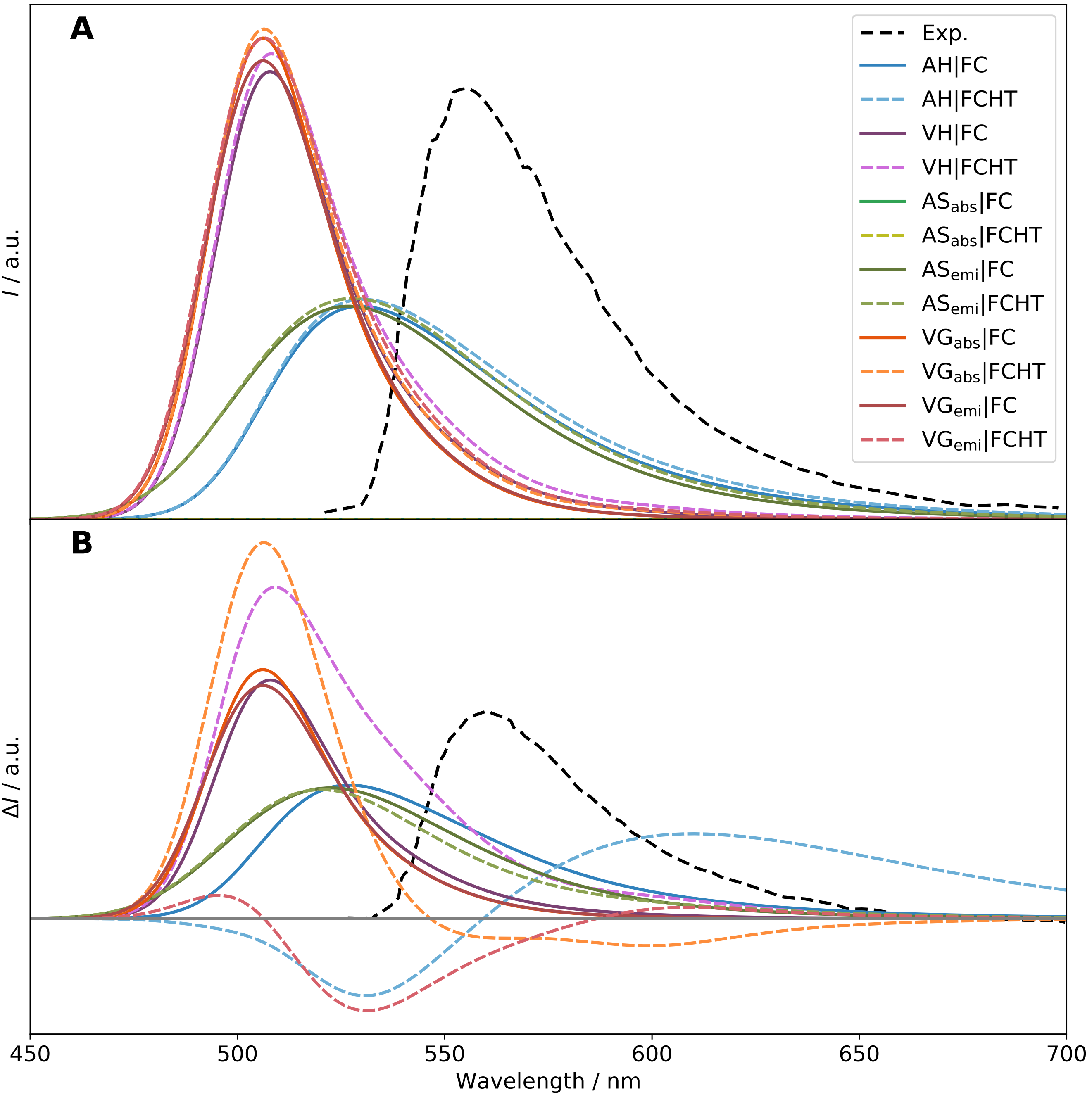

Figure S21. $S_1 \rightarrow S_0$ OPE (panel **A**) and CPL (panel **B**) of (*R*)-O-BODIPY with different models of vibronic spectra within the TD framework at T=298K. Gaussian distribution functions with half-widths at half-maximum of 500 cm^{-1} were used to simulate the experimental broadening.

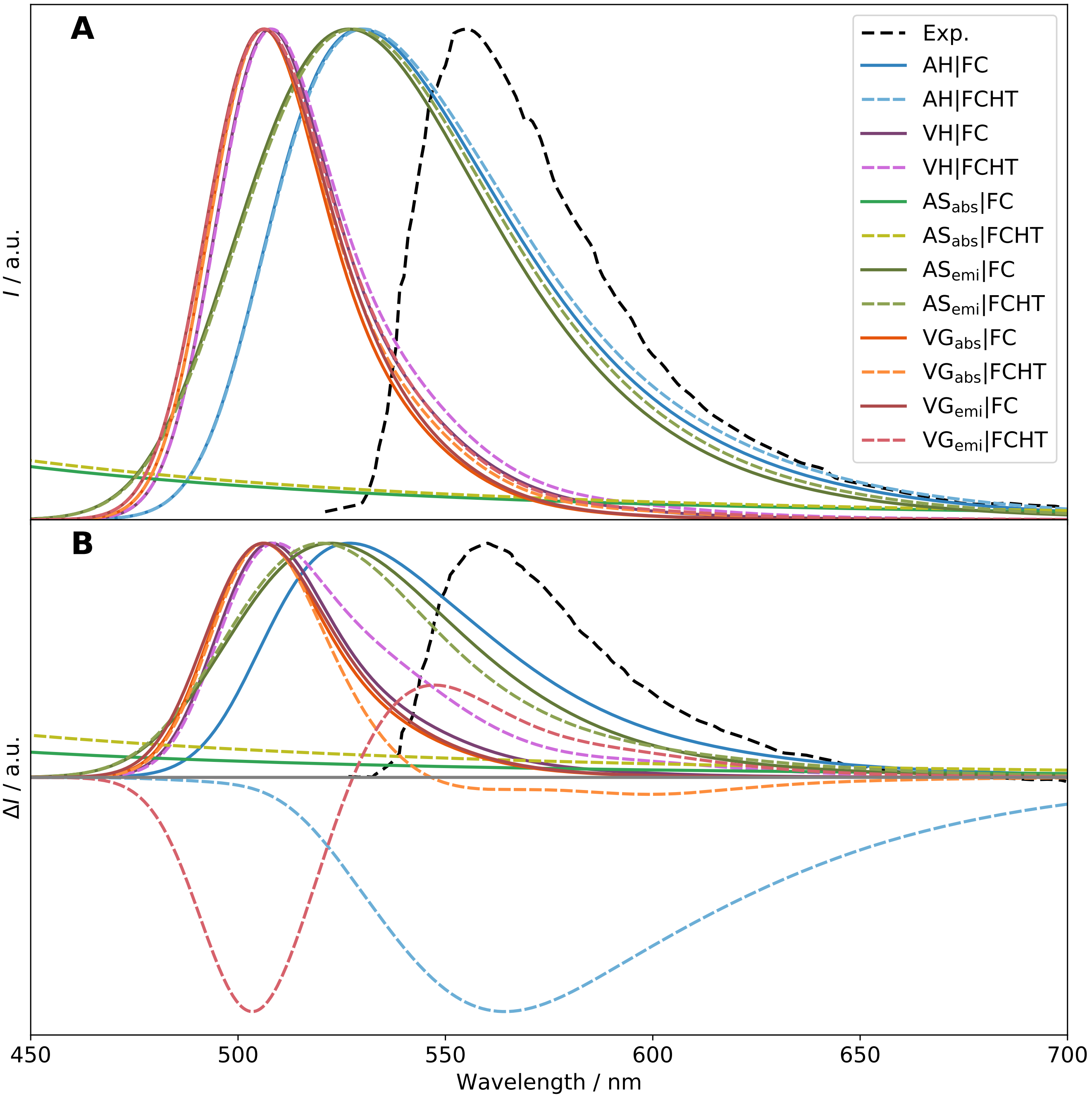

Figure S22. Normalized $S_1 \rightarrow S_0$ OPE (panel **A**) and CPL (panel **B**) of (*R*)-O-BODIPY with different models of vibronic spectra within the TD framework at T=298K. Gaussian distribution functions with half-widths at half-maximum of 500 cm^{-1} were used to simulate the experimental broadening.

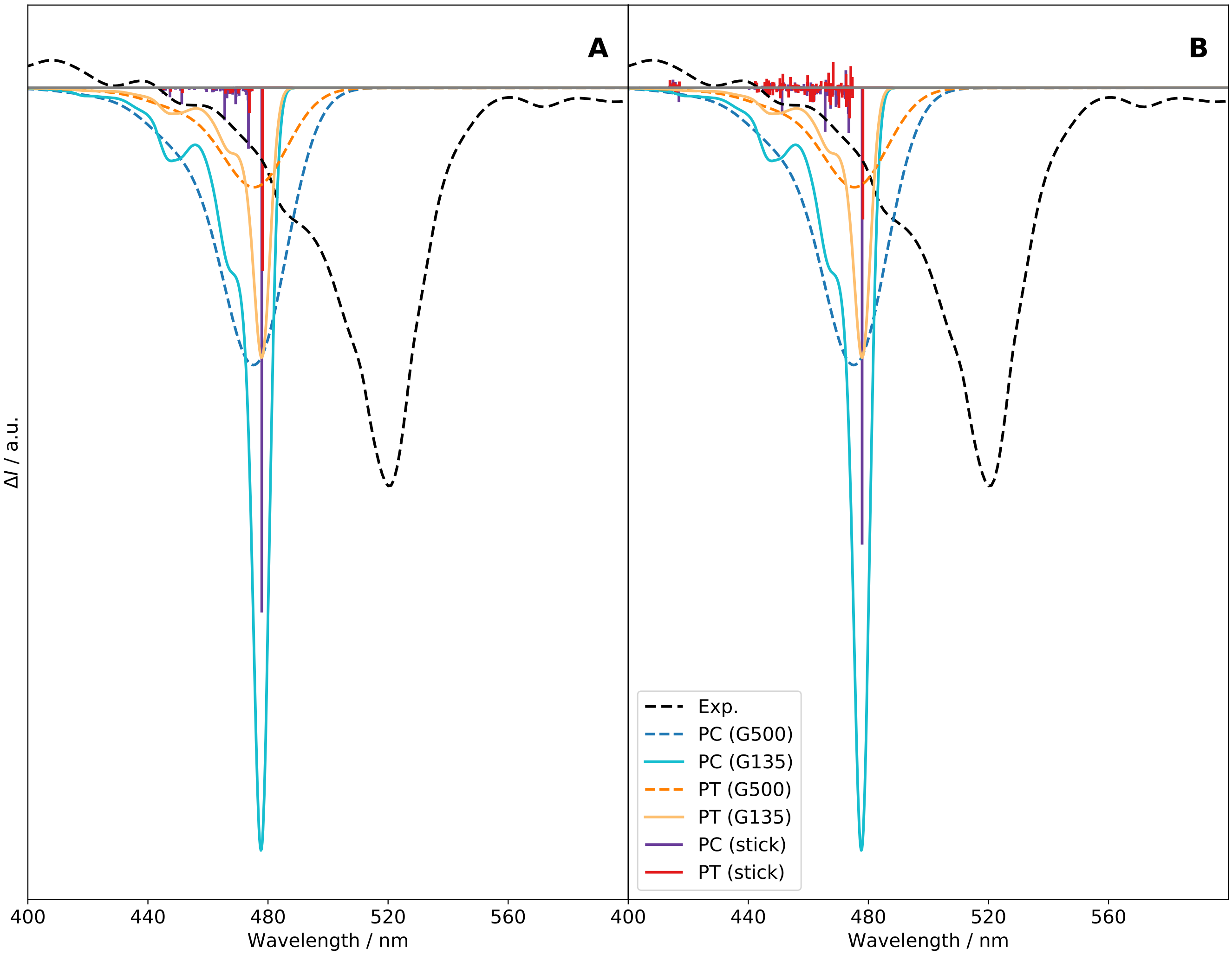

Figure S23. Vibronic $S_1 \leftarrow S_0$ ECD spectra of the PC and PT conformers of (*R*)-O-BODIPY computed at the TI VH|FC (panel **A**) and VH|FCHT (panel **B**) levels with different broadenings. Gaussian distribution functions are used with half-widths at half-maximum of 135 ("G135") and 500 cm^{-1} ("G500"). The stick spectra were arbitrarily scaled to fit in the figure.

REFERENCES

Bloino, J., Baiardi, A., and Biczysko, M. (2016). Aiming at an accurate prediction of vibrational and electronic spectra for medium-to-large molecules: An overview. *International Journal of Quantum Chemistry* 116, 1543–1574. doi:10.1002/qua.25188

Sharp, T. E. and Rosenstock, H. M. (1963). Franck–condon factors for polyatomic molecules. *The Journal of Chemical Physics* 41, 3453–3463. doi:10.1063/1.1725748
